# S100A proteins show a spatial distribution of inflammation associated with the glioblastoma microenvironment architecture

**DOI:** 10.7150/thno.100638

**Published:** 2025-01-01

**Authors:** Blanca Cómitre-Mariano, Berta Segura-Collar, Gabriel Velilla-Alonso, Rubén Contreras, Aurelio Hernandez-Lain, Manuel Valiente, Juan M. Sepulveda, Stephen Garrett Marcus, Guillermo García-Posadas, Luis Jiménez-Roldán, Ángel Perez-Nuñez, Ricardo Gargini

**Affiliations:** 1 Neurooncology Unit, Instituto de Investigación Biomédicas I+12, Hospital Universitario 12 de Octubre, Madrid 28041, Spain.; 2 Pathological anatomy, Hospital Universitario 12 de Octubre, Madrid 28041, Spain.; 3 Brain Metastasis Group, Spanish National Cancer Research Centre (CNIO), Madrid, Spain.; 4 Medical Oncology, Hospital Universitario 12 de Octubre, Madrid 28041, Spain.; 5 Cantex Pharmaceuticals, Weston, FL, USA.; 6 Neurosurgery, Hospital Universitario 12 de Octubre, Madrid 28041, Spain.

**Keywords:** Myeloid Cells, neuroinflammation, Glioblastoma, Alzheimer disease, clinical trials

## Abstract

**Background:** Glioblastoma IDH wild type (GBM IDH wt) has a poor prognosis and a strongly associated with inflammatory processes. Inflammatory molecules generate positive feedback with tumor cells fueling tumor growth as well as recruitment of immune cells that promote aggressiveness. Although the role of many inflammatory molecules is well known, there are many macromolecules, such as the S100A proteins, whose role is only now beginning to be established.

**Methods:** Using RNA-seq, bioinformatics tools and a cohort of glioma patients to validate the results, we have analysed the inflammatory processes involved in glioma. Transcriptional profiles were also used to define biological processes of relevance to specific S100A proteins. Finally, we characterized the relevant immune populations with an IHC analysis and transcriptional profiling.

**Results:** We have noted an increased expression of S100A in GBM IDH wt compared to gliomas IDH mutants. This allowed us to analyse the involvement of different members of the family, such as S100A9, A11 and A13 as possible regulators of inflammatory processes in the GBM-IDH wt microenvironment. Thus, we observed that S100A9 is located in hypoxic areas linked to the function of neutrophils, S100A11 is found in vascular areas associated with the function of perivascular pericytes and macrophages, and finally, S100A13 which is related to the dysfunction of microglia.

**Conclusion:** Our findings define different functions for S100A9, A11 and A13 proteins that are associated with the architecture of the glioblastoma microenvironment and define its progression. Moreover, these alterations can be reversed by the RAGE inhibitor, Azeliragon which is in a phase I/II clinical trial NCT05635734.

## Introduction

Gliomas represent the most prevalent and aggressive primary tumors of the central nervous system (CNS). In 2021 World Health Organization (WHO) classification, gliomas were stratified by tumor grade 1-4, using histological markers such as the presence of atypical and mitotic cells, vascular proliferation, and necrotic areas (1, 2). In addition, this recent classification also considers the presence of mutations in genes encoding the enzyme isocitrate dehydrogenase 1/2 (IDH1/2). IDH wild-type (IDH wt) gliomas are more aggressive compared to IDH mutant (IDH mut) variants 1, 2. Primary glioma subtypes include grade 2 and 3 IDH mut oligodendrogliomas, grade 2-4 IDH mut astrocytoma, and grade 4 IDH wt glioblastomas (GBM) 1, 2. GBMs are recognized as the most aggressive type of brain tumor, accounting for 48.6% of adult CNS malignancies 3. Standard therapeutic interventions include surgical resection followed by radiotherapy and chemotherapy with temozolomide (TMZ). However, the prognosis remains dismal, with a median survival of 15 months and only 5% of patients exceeding a 5-year survival threshold after diagnosis 4. Emerging therapeutic options, such as immunotherapy with PD-1/PD-L1 inhibitors, CAR-T cell therapy and anti-angiogenic drugs such as bevacizumab, present a new horizon of hope 5, 6. During gliomagenesis, significant mutations and genetic alterations accumulate alongside changes in the tumor microenvironment (TME), particularly vascular alterations and immune evasion 7. Distinctively, GBMs exhibit aberrant hypervascularization, with dilated, sinuous, and permeable blood vessels that accelerate their tumor progression 5. In addition, gliomas are markedly enriched with myeloid cells, specifically tumor-associated macrophages (TAMs) and myeloid-derived suppressor cells (MDSCs) 8. These cells, in synergy with tumor cells, release a multitude of inflammatory molecules, including cytokines and chemokines, which promote an immunosuppressive TME 8, 9. This environment favors and intensifies oncogenesis and tumor aggressiveness. However, the precise dynamics and underlying mechanisms driving these changes in glioma progression remain largely unknown 10.

Many inflammatory molecules have become desirable targets for new immunotherapies for refractory tumors 11. In recent years, the focus has shifted towards molecules that modulate the immune response within tumors, among which the members of the S100 family stand out 12. These molecules are characterized as calcium-binding and calcium-dependent sensor proteins, except for S100A10, and they present a characteristic helix-loop-helix structure with an EF-hand domain 5. The S100 family comprises at least 25 different members, including S100A, S100B, S100G, S100P and S100Z 5 with are exclusively expressed in vertebrates 5. Furthermore, these proteins are involved in a wide variety of functions, including maintenance of Ca^2+^ homeostasis, apoptosis, proliferation, migration, cell differentiation, angiogenesis and inflammation, acting as damage-associated molecular patterns (DAMPs) 5. Interestingly, their deregulated expression is associated with multiple diseases, including several types of cancer such as glioma (Supplementary Table 1)^12^-^14^. In the clinical practice, these proteins can be detected in various body fluids such as blood, urine, cerebrospinal fluid, sputum and faeces, suggesting their potential as molecular biomarkers for assessing glioma progression 12. Within this family, S100A9, S100A11 and S100A13 stand out and deserve special consideration. In this study, we observed an association of S100A proteins with different inflammatory agents that define the microenvironment of GBMs, such as necrotic, vascular and perivascular zones as well as infiltration zones governed by microglia. This complexity of the inflammatory microenvironment of GBMs defines it as one of the tumors most refractory to immunotherapies.

## Results

### IDH wt glioblastomas show immune infiltration associated with specific inflammatory patterns

**Figure 1:**
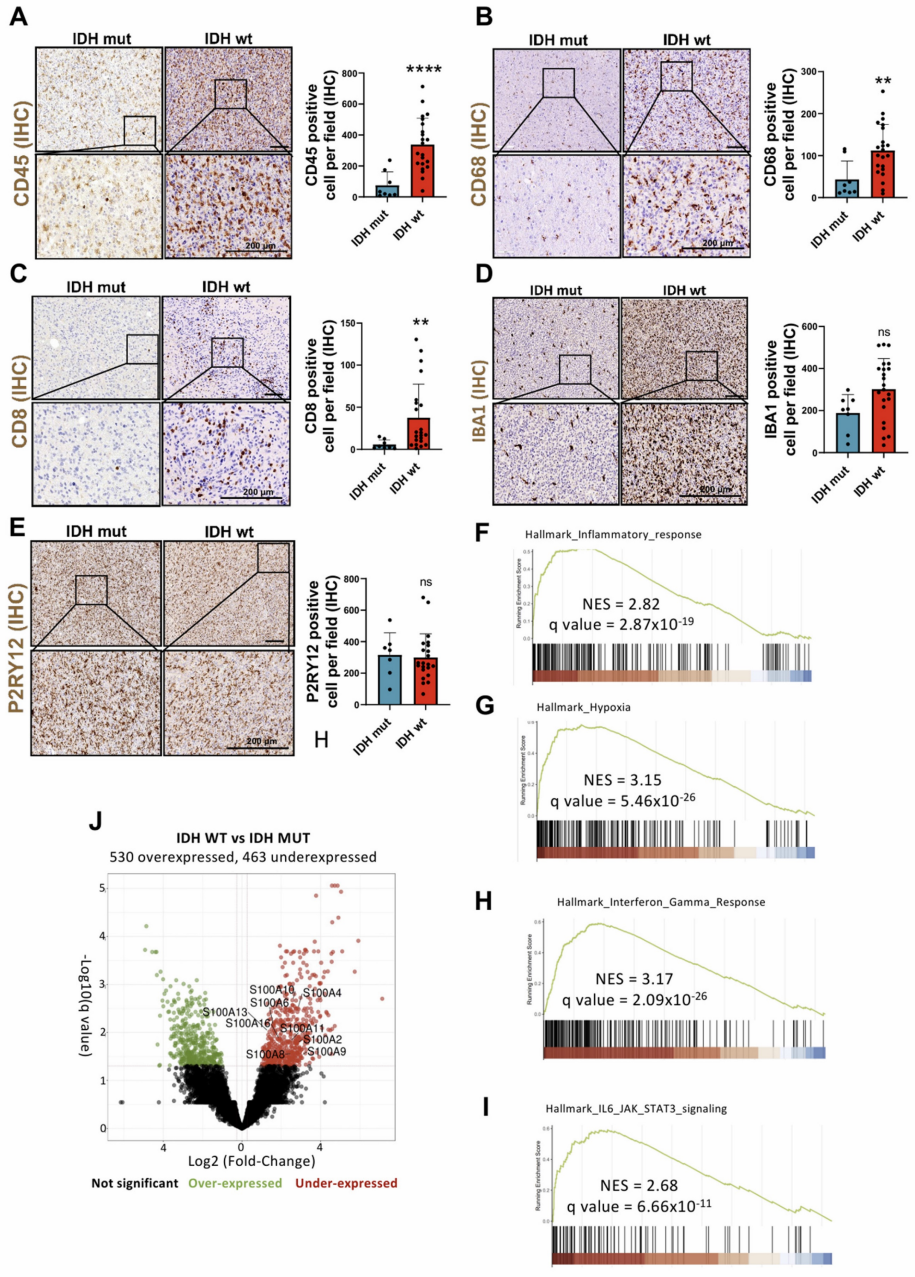
** Biological processes and proteins involved in the infiltration of immune cells of Glioblastoma IDH wt.** (**A-E**) Immune cell infiltration in IDH wt glioblastomas (n= 8) compared to IDH mut astrocytomas (n = 22) using IHC against CD45 (A), CD8 (B), CD68 (C) IBA-1 (D) and P2RY12 (E). Data represent mean ± SD. **** P ≤ 0.0001; ** P ≤ 0.01; n.s., not significant. Statistical significance was determined by Mann-Whitney test. (**F-I**) GSEA analysis using the differential expression of IDH wt compared to the IDH mutant shows inflammatory response (F), hypoxia (G), interferon gamma response (H) and IL-6/JAK/STAT3 signalling pathway (H), MSigDB database. (**J**) Volcano plot showing differential expression in IDH wt compared to the IDH mut cohort data obtained by RNA-seq (IlluminaHiSeq). Scale bar 200 μm (IHC).

Numerous studies have documented the involvement of the TME in the initiation and progression of cancer, in this sense, the brain chronic neuroinflammation is a critical factor in glioma tumorigenesis 15, 16. To understand the mechanisms through which the inflammatory response, derived from the infiltration of immune cells such as CD8 positive lymphocytes and CD68 positive macrophage into the brain, influences this disease we quantified the number of cells positive for CD45, CD68, CD8, IBA1 and P2RY12 in gliomas. The results showed a significant increase in the number of infiltrating leukocytes (CD45^+^), macrophages (CD68^+^), and T lymphocytes (CD8^+^) in IDH wt gliomas compared to IDH mut gliomas (**Figure 1A-C**). Meanwhile, no relevant change in the amount of microglia (IBA1^+^ and P2RY12^+^) was observed (**Figure 1D-E**). Then, we compared IDH wt vs. IDH mut gliomas using the expression data (Bulk RNA-seq) of all differentially expressed genes and subsequently performed an analysis through GSEA. This analysis showed a positive enrichment of the expression of various distinctive signatures, highlighting the inflammatory response, hypoxia, IL-6/JAK/STAT3 signalling and the response to gamma interferon (**Figure 1F-I**).

These molecular axes actively contribute to the creation of an immunosuppressive and pro-tumor TME through the release of inflammatory molecules, most notably endogenous DAMPs stand out. These molecular mediators not only promote the expansion and invasion of neoplastic cells, but also modulate the accumulation and infiltration of myeloid cells (GAM/TAM, TAN and MDSC), neoangiogenesis, suppression of antitumor immunity of T lymphocytes and therapeutic resistance (chemoresistance and radioresistance) 17-20. Among the multiple genes positively regulated in inflammatory processes, we find various members of the S100A family, such as S100A1, S100A2, S100A4, S100A6, S100A8, S100A9, S100A10, S100A11, S100A13 and S100A16 (**Figure 1J**), many of which act as DAMPs in glioma pathology 5, 17, 21.

### Expression profile of S100A genes in patients with gliomas and involvement of S100A9, S100A11 and S100A13 in the tumor microenvironment

**Figure 2:**
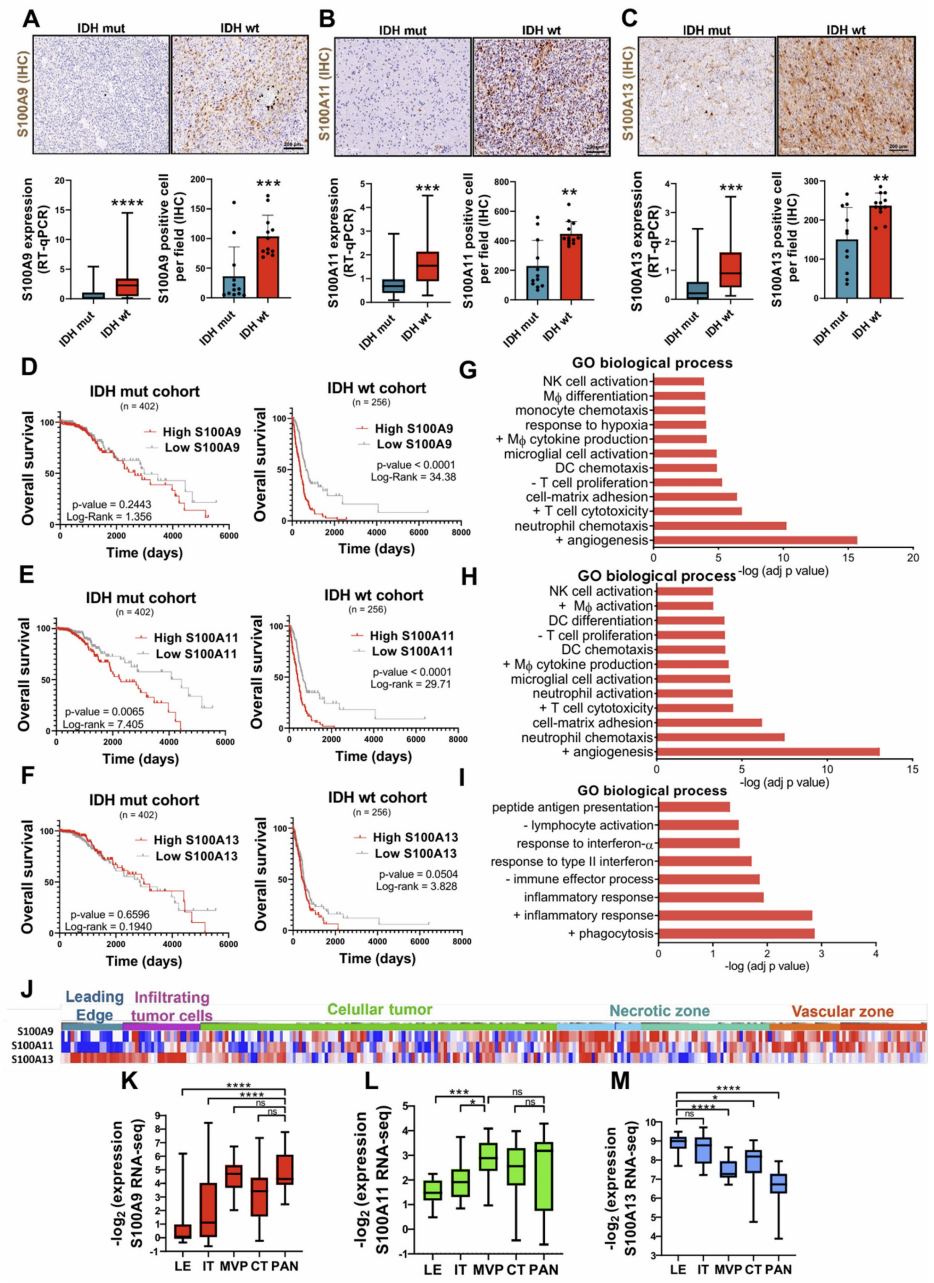
** Differential expressions of S100A9, S100A11 and S100A13 in various areas of the tumor drives TEM remodelling, correlating with lower survival of glioma patients.** (**A-C**) Representative images and expression levels at the transcriptomic (RT-qPCR) and protein (IHC) levels of S100A9 (A), S100A11 (B) and S100A13 (C) in our cohort of glioma patients classified into IDH mut (n = 22/12) and IDH wt (n = 42/12) respectively. Data represent mean ± SD. **** P ≤ 0.0001; *** P ≤ 0.001; ** P ≤ 0.01. Statistical significance was determined by the Mann-Whitney test. (**D-F**) Kaplan-Meier survival curves of patients with gliomas (TCGA LGG+HGG cohort), in relation to the expression of S100A9 (D), S100A11 (E), S100A13 (F). P values were determined by Mantel-Cox log-rank test. (**G-I**) Gene ontology enrichment analysis of pathways co-regulated with S100A9 (G), S100A11 (H), S100A13 (I) in the glioma cohort (TCGA GBM, Firehose Legacy). (**J-M**) Heat map (J) (IvyGAP) and quantification of the expression values (RNA-seq) of S100A9 (J), S100A11 (K), and S100A13 (L) in different tumor areas of the glioma (LE leading edge, IT infiltrating tumor cells, MVP microvascular proliferation, CT cellular tumor and PAN palisading cell around necrosis). Data represent mean ± SD. **** P ≤ 0.0001; *** P ≤ 0.001; * P ≤ 0.05; n.s., not significant. Statistical significance was determined by the One-way ANOVA ordinary test. +, positive regulation of; -, negative regulation of; NK cell, Natural Killer cell; DC Dendritic Cell; Mф, Macrophage. Scale bar 200 µm (IHC).

Comparative RNA-seq expression analysis between glioma patients, obtained from the astrocytoma IDH mut and GBM IDH wt cohorts from the TCGA database, showed significant overexpression of S100A2, S100A3, S100A4, S100A5, S100A6, S100A8, S100A9, S100A10, S100A11, S100A12 and S100A13 genes in IDH wt GBM compared to astrocytomas IDH mut (**Supplementary Figure 1A-B**), suggesting their possible relevance in the aggressiveness of this type of tumor. These results were corroborated, both at transcriptomic and protein level, in our own cohort of glioma patients (**Figure 2A-C**). Further analysis using single-cell RNA-seq (scRNA-seq) obtained through the Single-Cell Portal (GSE182109) 22, revealed the existence of distinctive expression patterns of different members of the S100A family. Specifically, S100A8 and S100A9 were expressed in myeloid cells, S100A10 and S100A11 in various cell types, especially in glioma tumor cells, and S100A13 and S100A16 in vascular cells (**Supplementary Figure 1C**). This result proposes distinct cellular phenotypes and differential functions for each of these genes.

Regarding the involvement of these proteins in the aggressiveness of the glioma, we identified that a high expression of S100A9 and S100A11 was associated with a significant decrease in the survival of patients with IDH wt tumors (TCGA LGG+GBM), suggesting a most unfavourable prognosis for these patients. Consequently, with our data S100A9 had been documented to have prognostic value in glioblastoma 23. However, this relationship was only relevant for S100A11 expression in IDH mut gliomas and no conclusive evidence was obtained regarding the prognostic role of S100A13 in tumor progression (**Figure 2D-F**). These findings suggest that S100A9 and S100A11 could be involved in regulating inflammatory processes related to glioma aggressiveness.

To investigate the biological processes in which S100A proteins of interest are involved in TME, a "David Gene Ontology" analysis was performed to examine the pathways co-regulated with these genes in glioma using TCGA GBM cohort. The results showed that the genes co-expressed positively with the different selected S100A genes could participate in a wide variety of key processes in tumor development, such as the immune response, angiogenesis or cell proliferation.

Specifically, S100A9 expression was linked to monocyte chemotaxis, macrophage differentiation, endothelial and T cell proliferation, and response to hypoxia (**Figure 2G**). While the other pathways associated with S100A11 expression were Dendritic cell (DC) differentiation, macrophage activation, lymphocyte proliferation, and integrin-mediated cell adhesion (**Figure 2H**). In contrast, S100A13 was exclusively correlated with immune processes related to inflammation, response to interferon, lymphocyte activation, presentation and processing of antigens, and phagocytosis (**Figure 2I**). Simultaneously, the histological distribution of the S100A genes of interest across the different regions of the tumor (leading edge, infiltrating tumor cells, cellular tumor, necrotic and vascular zone) was determined using the set of Ivy Glioblastoma Atlas Project (IvyGap). The results showed that S100A9 gene had a high expression in the necrotic and vascular areas of the tumor (identified by IHC staining of HIF-1α and CD34, respectively) (**Figure 2J, 2K**), while S100A11 was only representative in the vascular zones (**Figure 2J, 2L**) and S100A13 in the peripheral and infiltrating areas of the tumor (determined by GFAP IHC staining) (**Figure 2J, 2M**). These results were also corroborated in our own cohort of GBM patients by immunohistochemistry (**Supplementary Figure 2A-C**).

### Tumor peripheral microglial activation is linked to S100A13 expression

**Figure 3:**
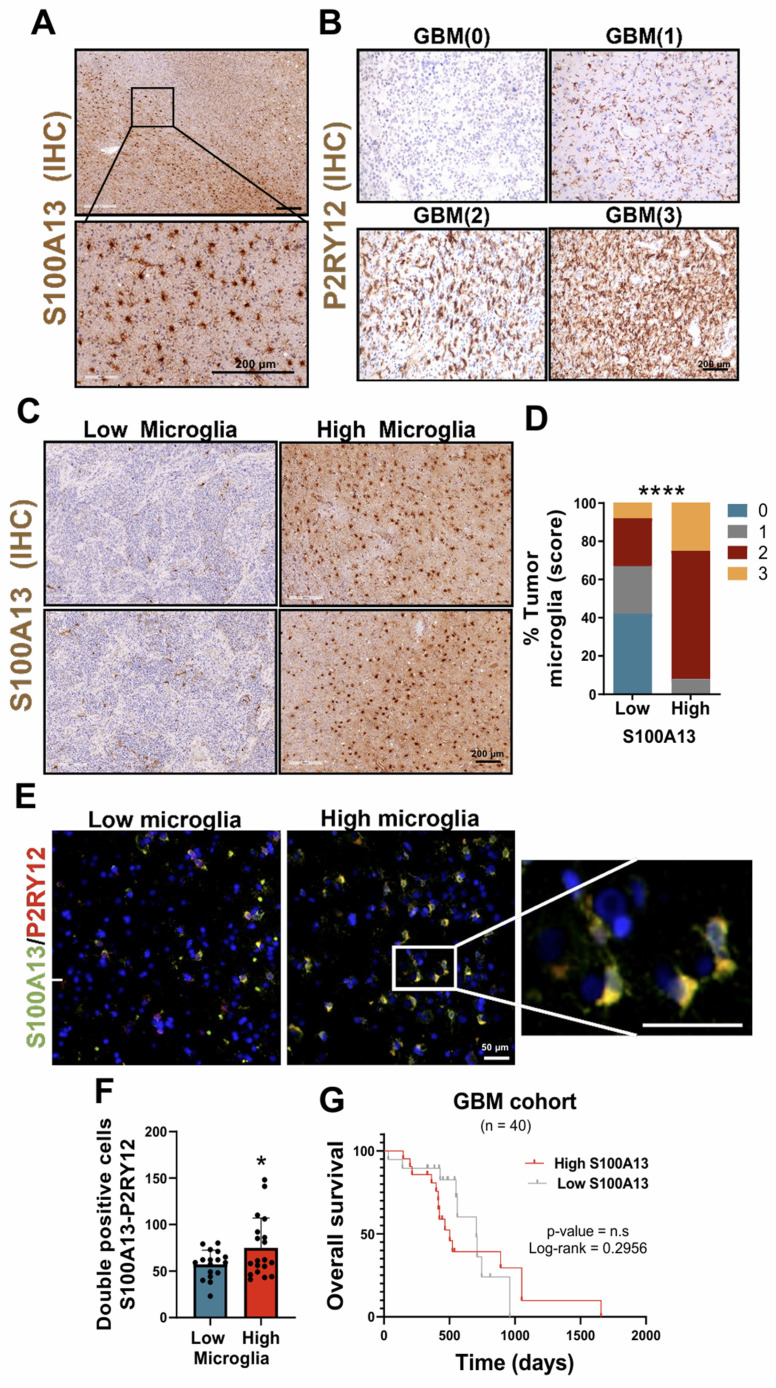
** S100A13 expression was associated with peripheral and infiltrating microglia in GBM.** (**A**) IHC images of S100A13 in the peripheral and infiltrating areas of the tumor. (**B**) IHC images of the P2RY12 in tumors with high and low microglia. (**C-D**) Representative images of IHC (C) and quantification (D) of S100A13 in high and low microglia tumor sections. (**E-F**) IF images (E) and quantification (F) of S100A13^+^ P2RY12^+^ double-positive cells in high (n = 4) and low (n = 4) microglia sections. Data represent mean ± SD. **** P ≤ 0.0001; * P ≤ 0.05. Statistical significance was determined by Student's *t*-test(**G**) Kaplan-Meier survival curve of our cohort of GBM patients (n = 40) stratified based on S100A13 expression.; n.s., not significant. P values were determined by Mantel-Cox log-rank test. Scale bar 200 μm (IHC) and 50 µm (IF).

The study on the involvement of S100A13 in the development of GBM revealed that the cells expressing this gene were located in peripheral and infiltrating areas of the tumor. In addition, these cells presented a distinctive morphology with long and irregular processes characteristic of microglia (resident immune cells of the brain) (**Figure 3A**). For this analysis, the tumor samples were stratified into high and low microglia content, using a score range from 0 to 3 depending on the presence of different amounts of these cells determined by immunohistochemical (IHC) staining with the microglial marker P2RY12 (**Figure 3B**). An increase in the number of S100A13 positive cells was found in tumors with high microglia content compared to those GBM with lower numbers of microglial cells (**Figure 3C-D**).

Next, to determine the cell type that expressed S100A13, the public data from a scRNA-seq study of patients with gliomas obtained through the Single-Cell Portal (GSE182109) were used 22. The results showed that S100A13 was expressed in myeloid cells, co-expressing with microglial markers such as IBA1/AIF1, P2RY12 and TREM119, although in a less prominent manner. After performing immunofluorescent (IF) co-staining with the marker P2RY12 in samples from patients with glioma, we identified that the cells that expressed S100A13 corresponded to microglia (**Figure 3E-F**), capable of actively contributing to the neuroinflammation of the GBM due to their location in the leading edge of the tumor. Furthermore, a greater presence of S100A13^+^ and P2RY12^+^ microglia was observed in those GBM with high microglia compared to those with low microglia. Finally, we confirmed in our own cohort of patients with GBM IDH wt that there was no significant correlation between the expression of S100A13 and the survival of these patients (**Figure 3G**).

### Tumor hypoxia related to S100A9 expression favours the localization of MDSC in necrotic areas of GBM

**Figure 4:**
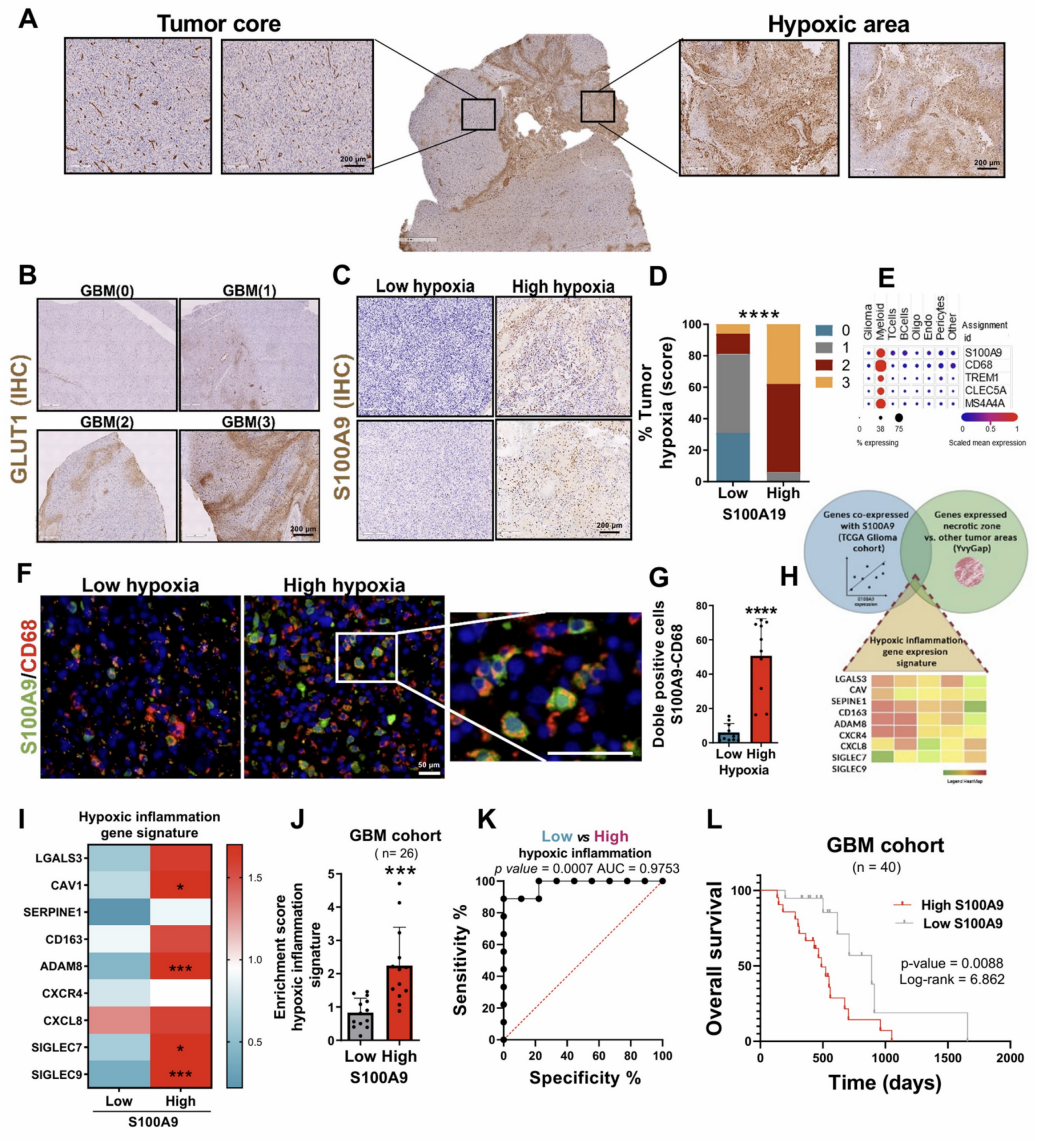
** S100A9 expression was related to hypoxic inflammation in necrotic areas of GBM. (A-B)** IHC images of GLUT1 in areas with high and low hypoxia. (C-D) Representative IHC images (C) and quantification (D) of S100A9 in high- and low-hypoxic tumor sections. (**E**) scRNA-seq analysis of expression levels of myeloid markers (CD68, TREM1, CLEC5A, MS4A4) in glioma. **(F-G)** IF images (F) and quantification (G) of S100A9^+^ CD68^+^ double-positive cells in high (n = 5) and low (n = 5) hypoxic glioma sections. Data represent mean ± SD. **** P ≤ 0.0001**.** Statistical significance was determined by Student's t-test.** (H)** Venn diagram of the intersection of genes co-expressed with S100A9, related to inflammatory processes and expressed in the hypoxic and necrotic zone of the tumor (*hypoxic inflammation signature).* **(I-J)** Heat map (I) and quantification (J) of *hypoxic inflammation signature* expression in our own cohort of GBM patients (n = 26) stratified based on S100A9 expression. Data represent mean ± SD. *** P ≤ 0.001. Statistical significance was determined by Student's t-test. **(K)** ROC curve to evaluate the diagnostic efficacy of *hypoxic inflammation gene signature* expression in GBM. **(L)** Kaplan-Meier survival curve of our GBM patient cohort (n = 40) stratified based on S100A9 expression. P values were determined by Mantel-Cox log-rank test. Scale bar 200 µm (IHC) 50 μm (IF).

Based on the results obtained previously, the first objective was to validate the specific expression of S100A9 in the hypoxic and necrotic areas of the tumor (**Figure 2G**). Several previous studies have already demonstrated a strong relationship between these cellular phenomena, where the oxygen deprivation in the TME (hypoxic TME) induces unprogrammed cell death (necrosis) 24, 25. Thus, the samples were stratified into high and low hypoxia, using a score from 0 to 3 depending on the presence of these regions, determined by IHC staining with the hypoxic and glycolytic marker GLUT1 (**Figure 4A-B**). We observed S100A9-positive cells in the hypoxic and necrotic areas of tumors (measured by IHC, **Figure 4C**), with significantly greater infiltration of these cells in GBM classified as high-hypoxia versus low-hypoxia tumors (**Figure 4D**).

Next, to characterize the cell type that expressed S100A9, we again used public data from the previously cited single-cell scRNA-seq study (GSE182109, M&M) 22. The results showed that S100A9 was expressed in an immunosuppressive myeloid population, due to its co-expression with markers such as TREM1, CLEC5A and MS4A4A (**Figure 4E**). The histological co-staining with CD68 confirmed that those cells expressing S100A9 corresponded to myeloid cells (**Figure 4F**), which play a relevant role in the inflammation and tumorigenesis associated with GBM, as we have recently demonstrated 17, 26-28. In addition, a greater infiltration of S100A9^+^ and CD68^+^ MDSCs was also observed in areas with high hypoxia compared to those with low hypoxia (**Figure 4G**).

Finally, to determine the inflammatory process associated with the expression of S100A9, we defined an inflammation gene signature composed of those genes co-expressed with S100A9, which were implicated in inflammatory processes (TCGA GBM cohort) and upregulated in the hypoxic and necrotic tumor areas (IvyGAP) (**Figure 4H**), called *hypoxic inflammation* signature. It was confirmed that the overexpression of *hypoxic inflammation* signature occurred in those GBM with a high content of CD68^+^/S100A9^+^ cells compared to those with low cell infiltration (**Figure 4I-J**). Furthermore, ROC curve analysis showed a high percentage of sensitivity and specificity, with an AUC of 0.9753, indicating that the expression of the *hypoxic inflammation* signature is a powerful clinical and diagnostic marker for GBM (**Figure 4K**). Finally, as expected, GBM patients who showed high expression of S100A9 had a significantly lower survival than those with low gene expression (**Figure 4L**).

Together, these results demonstrated that hypoxia drives a toxic inflammatory state in the TME of the GBM, linked to the infiltration of S100A9^+^ MDSCs. The presence of these cells favors an immunosuppressive and protumoral TME promoting the aggressiveness of the GBM.

### Perivascular inflammatory profile of S100A11 and its association with GBM proliferation and recurrence

**Figure 5:**
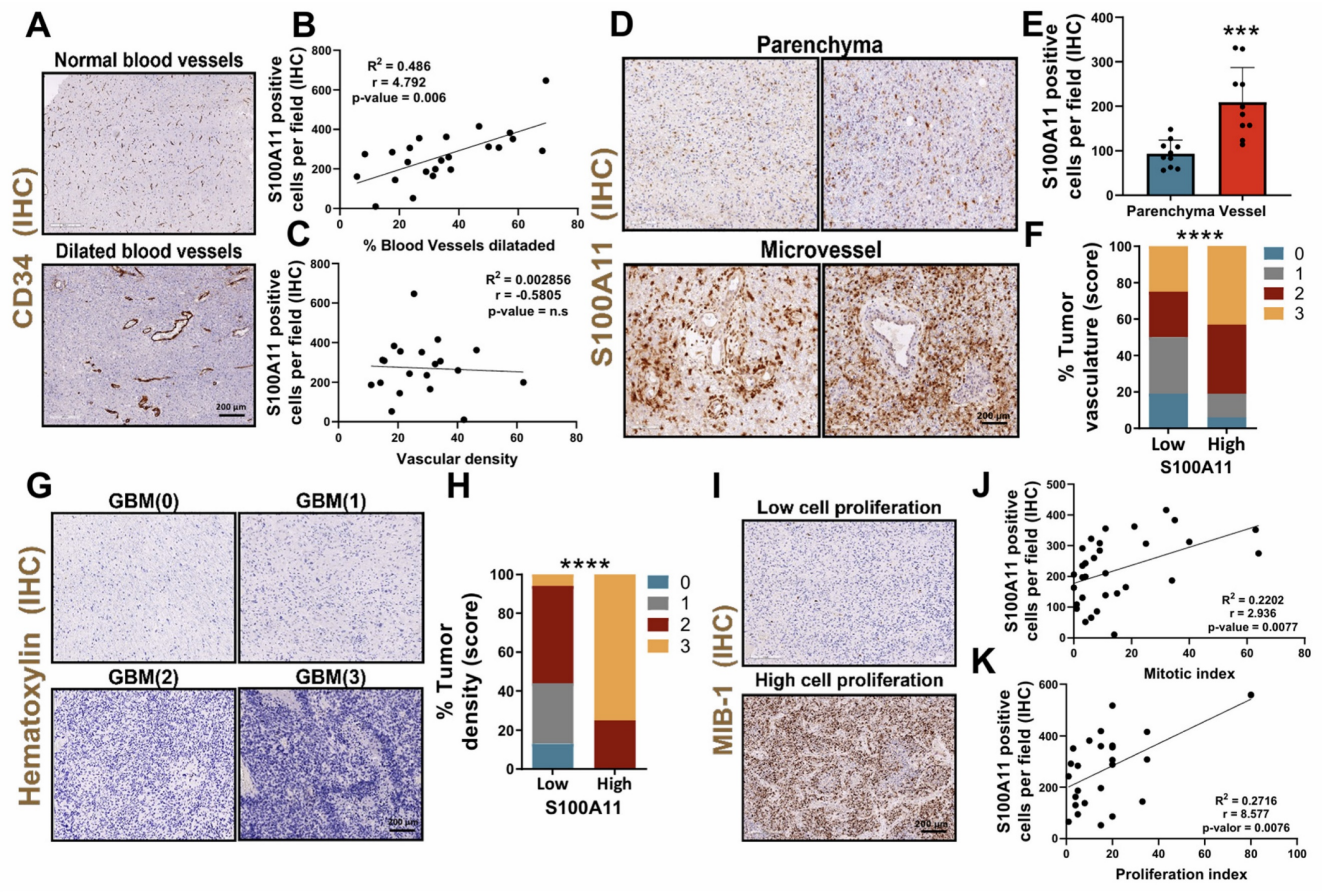
** S100A11 expression was associated with perivascular inflammation, linked to GBM proliferation.** (**A**) IHC images of CD34 in tumor sections. (**B-C**) Correlation analysis between S100A11 expression and percent of dilated vessels (n = 23) (B) and vascular density (n = 20) (C). Data represent mean ± SD. Statistical significance was determined by Pearson's rank correlation (**D-E**) Representative IHC images (D) and quantification (E) of S100A11-positive cells in the brain parenchyma and around blood vessels. Data represent mean ± SD. *** P ≤ 0.001. Statistical significance was determined by Student's t-test. (**F**) Quantification of S100A11^+^ cells in tumor sections with high and low vasculature. Data represent mean ± SD. *** P ≤ 0.001. Statistical significance was determined by Student's t-test. (**G**) Representative IHC images of hematoxylin eosin staining tumor areas. (**H**) Quantification of S100A11^+^ cells in sections with high and low tumor density. (**I**) IHC images of MIB-1 in tumor sections. (**J-K**) Correlation analysis between the number of S100A11^+^ cells and the mitotic (K) and proliferative (L) index. Data represent mean ± SD. Statistical significance was determined by Pearson's rank correlation Scale bar 200 μm (IHC).

Currently, the underlying mechanisms of the perivascular inflammatory process associated with GBM remain unknown, despite being one of the main causes associated with progression, so we studied the role of S100A11 in this context. To address this, we performed a vascular density and number of dilated vessels analysis quantified employing IHC with the endothelial marker CD34 (**Figure 5A**). Histological analysis of this marker also allowed stratification of the samples into high and low tumor vascularity, using a score from 0 to 3 according to the presence of dilated vessels. We observed a positive correlation between S100A11 expression and the percentage of dilated vessels in patients with GBM (**Figure 5B**), while it was not significant for vascular density (**Figure 5C**). In more detail, a relevant increase in S100A11 expression was identified around the vessels compared to the brain parenchyma (**Figure 5D-E**). In addition, a significantly higher presence of S100A11 positive cells was confirmed in GBMs classified as high vasculature/vasodilated tumors versus low vasculature/vasodilated tumors (Figure 5F). These findings indicate that S100A11 expression could favour deregulation in the tumor vasculature, which ultimately drives tumor progression. To validate this hypothesis, the samples were stratified into high and low cell density groups, using a score from 0 to 3 (**Figure 5F**), determined by Haematoxylin-Eosin staining. A significant increase in hypercellular areas was observed in GBM with high expression of S100A11 (**Figure 5G-H**), and there was a positive correlation between S100A11 expression and the proliferative and mitotic index, as measured by IHC with the tumor marker MIB-1 (**Figure 5I-K**). These results confirm that S100A11 expression in perivascular areas correlates with increased vasodilation and proliferation in GBMs.

**Figure 6:**
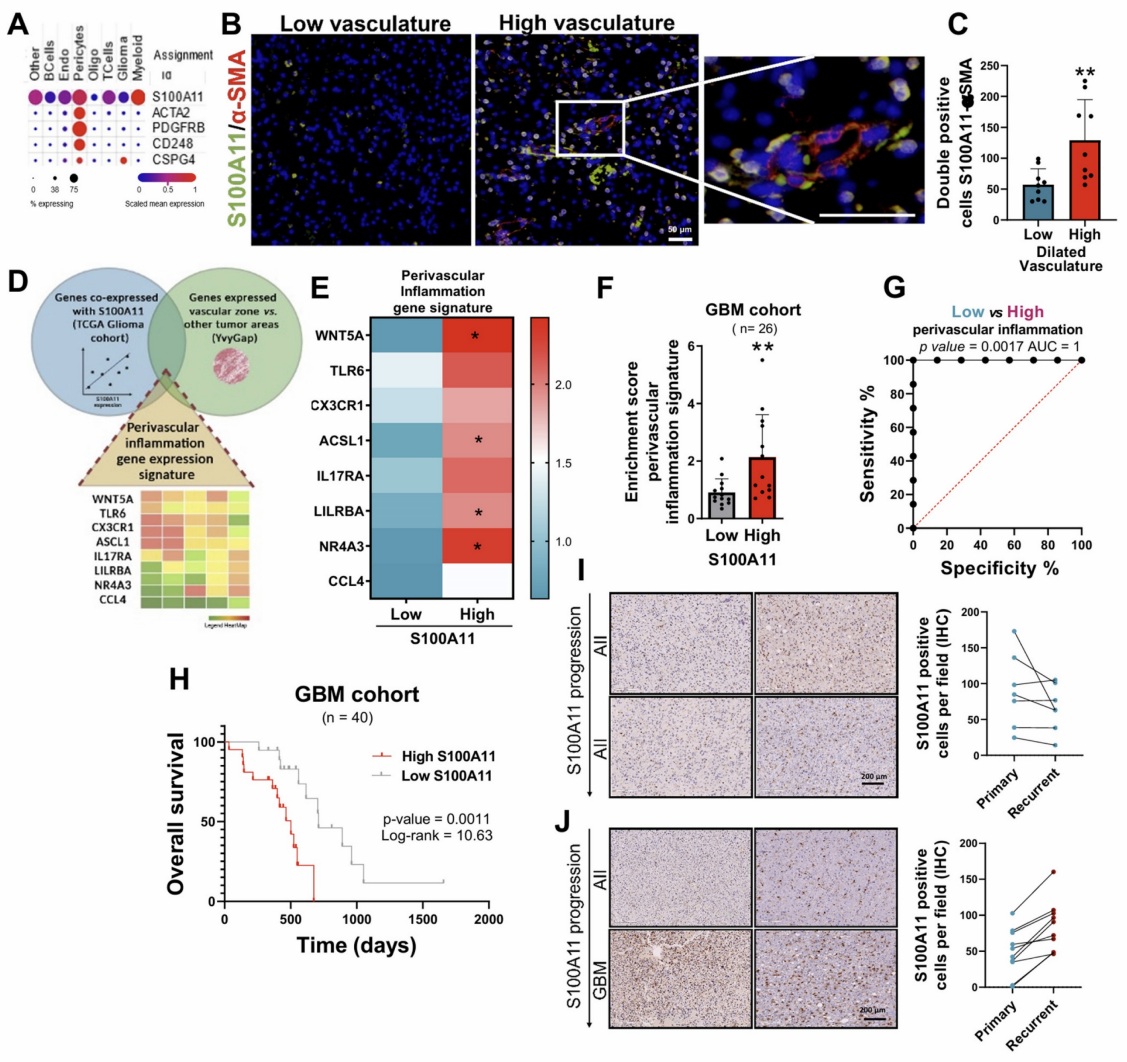
** The expression of S100A11 was associated with pericyte cells, and their perivascular inflammation, linked to poor prognosis and glioma recurrence.** (**A**) scRNA-seq analysis of the expression levels of S100A11 and vascular markers (ACTA2/ αSMA, PDGFRB, CD348, CS248) in gliomas. (**B-C**) IF images (B) and quantification (C) of S100A11^+^ αSMA^+^ double-positive cells in tumor sections with high (n = 3) and low (n= 3) vasculature. Data represent mean ± SD. ** P ≤ 0.01. Statistical significance was determined by Student's t-test. (**D**) Venn diagram of the intersection of genes co-expressed with S100A11 related to inflammatory processes and expressed in the vascular zone (*perivascular inflammation signature*). (**E-F**) Heat map (E) and quantification (F) of *perivascular inflammation signature* expression in our own cohort of GBM patients (n = 26). Data represent mean ± SD. ** P ≤ 0.01. Statistical significance was determined by Student's t-test. (**G**) ROC curve to evaluate the diagnostic efficacy of the *perivascular inflammation genetic signature* in GBM. (**H**) Kaplan-Meier survival curve of the GBM patient cohort (n = 40) stratified by S100A11 expression. P values were determined by Mantel-Cox log-rank test. (**I-J**) Representative IHC images of S100A11 from paired glioma samples (primary and recurrent tumor) with progression (I) and without progression (J). Scale bar 50 μm (IF).

Subsequently, to determine the identity of those cells that expressed S100A11, the data from the scRNA-seq study (GSE182109) was used again 22*.* The results show that S100A11 was expressed in different cell types, highlighting its possible vascular identity due to its co-expression with vascular markers such as ACTA2/α-SMA, PDGFRβ, CD248 and NG2/CSPG4 (**Figure 6A**). Histological co-staining of this protein with α-SMA (**Figure 6B**), identified that those cells that expressed S100A11 could correspond to tumor cells with vascular function, which play a relevant role in the regulation and remodeling of the TME of the glioma, as our group has previously described 7. In addition, a greater infiltration of these S100A11^+^ and α-SMA^+^ cells were also observed in areas with high dilated blood vessels compared to tumors with fewer of these (**Figure 6C**). Next, to study the inflammatory process related to the expression of S100A11, a genetic signature of inflammation was characterized, composed of those genes co-expressed with S100A11 (TCGA GBM cohort), which were linked to inflammatory processes and upregulated in the tumor vascular zone (using IvyGAP dataset) (**Figure 6D**), called *perivascular inflammation* signature. This signature was overexpressed in those GBM with high expression of S100A11 compared to those with low expression of this gene (**Figure 6E-F**). Likewise, the ROC curve analysis revealed a high sensitivity and specificity, with an AUC of 1, which highlights the high efficacy of the expression of the *perivascular inflammation* signature as a highly effective diagnostic biomarker for GBM, outperforming the *hypoxic inflammation* signature (**Figure 6G**). GBM patients with high expression of the *perivascular inflammation* signature had significantly lower survival rates compared to those with low expression (**Figure 6H**). Together, these findings show that S100A11 expression and vascular dysregulation are closely linked to a type of inflammation that promotes glioma tumor progression.

Finally, the involvement of S100A11 expression of pathology progression was performed, comparing primary tumors with recurrences, in our own cohort of recurrent gliomas. Thus, an increase in S100A11 expression was found in those tumors that progressed towards a more aggressive phenotype (**Figure 6I**), while it was not observed in samples without changes in histological classification (**Figure 6J**). These findings suggest that S100A11 overexpression plays a crucial role in glioma recurrence.

Taken together, our results allow us to propose the existence of different inflammatory profiles associated with the expression of S100A genes in GBM. According to this idea, gliomas could be classified according to a *hypoxic inflammation* linked with S100A9 expression, a *perivascular inflammation* related to S100A11 expression, and a microglial inflammation associated to S100A13 expression.

### Perivascular and hypoxic inflammation-dependent response to Azeliragon treatment in GBM

Two recent findings in the clinical setting of brain metastases have raised the importance of S100A genes in development and radioresistance 23, 29. To explore the potential therapeutic role of GBM inflammation, we investigated the therapeutic effect of Azeliragon (AZG, TTP488, PF-04494700), a small molecule in clinical trials that inhibits the binding of ligands, including S100 proteins, to the receptor for advanced glycation end products (RAGE). Although this drug was initially developed for the treatment of Alzheimer's and crosses the blood-brain barrier (BBB) with good tolerance and pharmacological safety, a therapeutic effect in Alzheimer's disease was not identified in phase III clinical trials 30. Currently, AZG is being used in phase I/II clinical trials in combination with chemoradiotherapy for the treatment of patients with GBM in different hospitals such as the Hospital 12 de Octubre where we collaborate (NCT05635734). This therapeutic strategy is based on the previous identification of S100A9 as a relevant biomarker of radioresistance in liquid biopsies 21.

**Figure 7:**
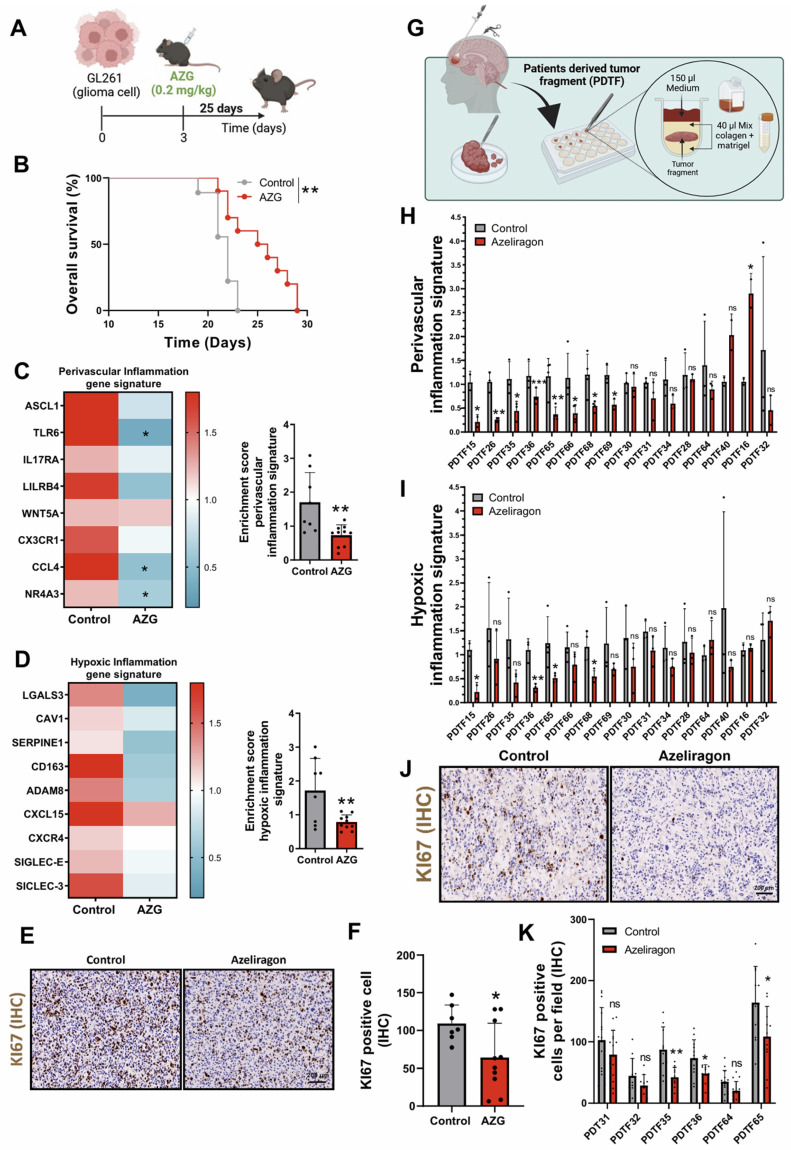
** Response associated with perivascular and hypoxic inflammation with treatment with Azeliragon in glioblastoma mouse model and PDTFs model.** (**A**) Schematic diagram of AZG treatment in mice injected with GL261 tumors. (**B**) Kaplan-Meier overall survival curves of control and AZG-treated mice. GL261 control (n = 9) and AZG (n = 10). ** P ≤ 0.01 by Mantel-Cox log-rank test for survival experiments. (**C-D**) Heatmap and quantification of perivascular (C) and hypoxic (D) inflammation gene signature after treatment with AZG (0.2 mg/kg for 25 days). (**E-F**) Representative IHC images (E) and quantification (F) of KI67 in murine model. Data represent mean ± SD. ** P ≤ 0.01; * P ≤ 0.05. Statistical significance was determined by Student's t-test. (**G**) Schematic representation of the PDTF procedure. (**H-I**) Quantification of the gene expression signature of perivascular (H) and hypoxic (I) inflammation after treatment with AZG (10 µg/ml for 24 h). (**J-K**) Representative IHC images (J) and quantification (K) of KI67 in PDTFs. Data represent mean ± SD. *** P ≤ 0.001; ** P ≤ 0.01; * P ≤ 0.05; n.s., not significant. Statistical significance was determined by Student's t-test. PDTF; Patients Derived Tumor Fragment; AZG; Azeliragon.

To evaluate the therapeutic effect of AZG on GBM, we performed a parallel preclinical study by implanting GL261 cells into the brain of C57BL/6 mice. From day three of tumour implantation, mice were treated daily with AZG at a dose of 0.2 mg/kg for 25 days (**Figure 7A**). The results showed an increased survival rate of AZG-treated mice compared to controls (**Figure 7B**) 31. Additionally, AZG-treated mice showed a notable reduction in both *perivascular* and *hypoxic inflammatory signatures* (**Figure 7C-D**). To further investigate the pharmacological effect of AZG in this murine GBM model, IHC staining for the tumor proliferation marker KI67 was performed, revealing a marked decrease in cell proliferation in the AZG-treated mice (**Figures 7E-F**).

Finally, to translate the results into clinical settings, we developed an organotrophic culture platform using patient-derived tumor fragments (PDTFs), as previously described in Voabil *et al.* 2021 32. In this platform, fresh tumor tissue from GBM patients was treated with AZG (10 µg/ml) for 24 hours to evaluate the anti-inflammatory response using the genetic signatures of *hypoxic* and *perivascular inflammation* (**Figure 7G**). These results showed that AZG generates a significant reduction in both signatures, *perivascular inflammation* (**Figure 7H, Supplementary Figure 3**) and *hypoxic inflammation* (**Figure 7I, Supplementary Figure 4**) compared to the control condition. Strikingly/Notably AZG generated a significantly greater decrease in the number of tumor responders with the *perivascular inflammation* signature (8 of the 16 tumors analysed, **Supplementary Figure 5A**) compared to those with *hypoxic inflammation* signature (4 of the 16 tumors analysed **Supplementary Figure 5B**). Furthermore, PDTFs exhibiting reduced inflammatory signatures also showed a marked decrease in cell proliferation (**Figures 7J-K**).

These findings provide clear evidence of the existence of diverse GBM patients' subgroups based on a specific inflammatory profile. They also underscore the potential of AZG as a treatment for GBM. Moreover, the use of PDTF models provides a valuable platform for identifying patients who are likely to respond to treatment, based on key biomarkers such as the proteins S100A11 and S100A9 and their associated inflammatory genetic signatures. This approach could enable the development of more personalized therapies, targeting particular inflammatory profiles in different GBM patient subgroups.

## Discussion

**Figure 8:**
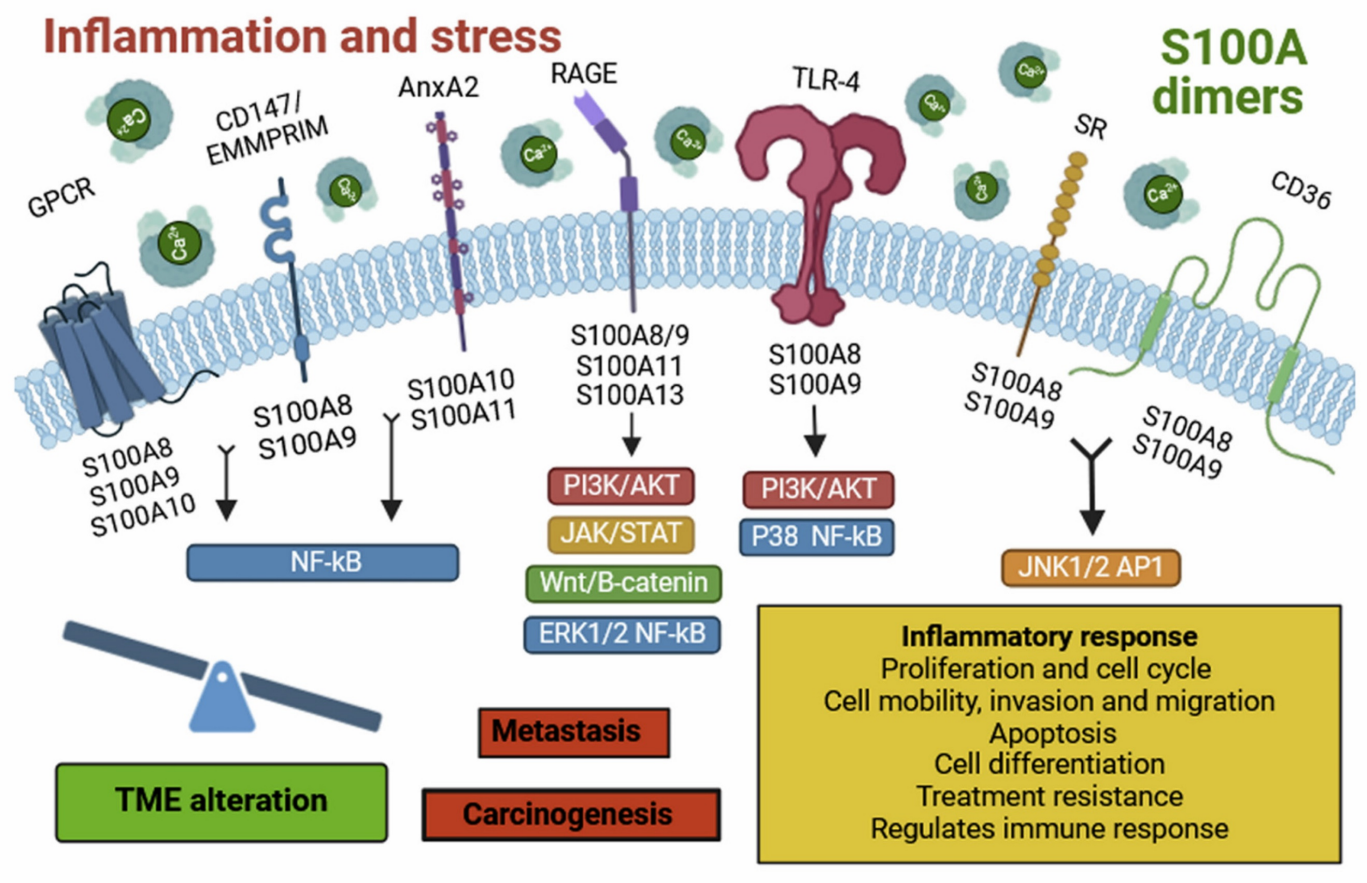
Representative diagram of the extracellular S100s signaling.

Inflammatory processes are associated with multiple diseases and are often considered as potent drivers that stimulate or even trigger pathologies. One of the best examples is tumorigenesis, which has been shown to depend on inflammation to initiate, develop and progress. Thus, understanding the inflammatory process has garnered significant interest because of its involvement in tumor development and its impact on the effectiveness of cancer immunotherapies. In the special case of GBMs, the tumor development is masked within a neuroinflammatory process that frequently is associated with the patient's poor prognosis 7. The cell composition of the GBM microenvironment has been the target of multiple therapies, including at the level of vascular and immune cells 7, 33. Our group has even shown that there is a connection between vascular molecules with inflammatory processes in GBMs, which can be inhibited with VEGFA (Vascular endothelial growth factor, associated with angiogenesis) blockers, such as bevacizumab 28. For a deeper understanding of the neuroinflammatory processes, it's crucial to highlight the sterile inflammation processes, which can be of great relevance for pathologies of the central nervous system. These processes encompass a wide variety of DAMP-sensing receptors such as TLR-4 or RAGE, which are usually deregulated in cancer and may contribute to gliomagenesis 12-14, 34. In addition, RAGE is transmembrane protein stands out for being key in the initiation and maintenance of the inflammatory response in various pathologies, including neurodegenerative and cardiovascular diseases, diabetes and different types of cancer 35, 36. Among the multiple endogenous ligands of this receptor, we found on the target proteins investigated in this article: S100A9, S100A11 and S100A13 37,38, specifically selected for this reason (**Figure 8**).

Despite our limited understanding of the pathological function of S100A proteins in the GBM microenvironment, their therapeutic potential remains an area of ongoing investigation. Here we show and dissect the differential functions of S100A which are associated with specific processes of the architecture of the glioblastoma microenvironment.

On the other hand, S100A9 is expressed in various cell types, such as monocytes, neutrophils, dendritic cells (DCs), macrophages, fibroblasts, tumor cells, endothelial cells, keratinocytes, and glioma stem cells (GSCs) 5, 10. In the case of GBM, expression is restricted to the myeloid population, which has been observed by other authors and we have validated that it is normally found in the hypoxic zone 28, 39, 40. This localization can be contributed to a pro-tumor immune microenvironment, characterized by a dysfunctional immune phenotype, such as that seen in MDSCs 28. These cells are known for their highly suppressive activity on the immune response and have also been implicated as inducers of GBM aggressiveness, especially in males 28. S100A9 localizes to the plasma membrane, intermediate filaments, or cytosol (10). This protein exhibits an immunosuppressive phenotype 41 and can bind to receptors such as RAGE, TLR-4, SR, GPCR, CD36, CD147, and EMMPRIM 5. Its interaction activates multiple signalling pathways such as NF-κB, MAPK, STAT3, and PI3K/AKT/mTOR, leading to the release of inflammatory cytokines and chemokines. These molecules contribute to the establishment of an inflammatory TME, favouring tumorigenesis, metastasis, angiogenesis, tumor progression, and drug resistance 42. Under pathological conditions, S100A9 is secreted by necrotic or immune-activated cells and plays a crucial role in the chemotaxis and infiltration of monocytes, TAMs, and tumor-associated neutrophils (TANs) in the tumor region. Consequently, these cells suppress the antitumor immune response, facilitating tumorigenesis and metastasis 41, 43.

In response to inflammation and stress, inflammatory cells such as macrophages, lymphocytes, neutrophils and fibroblasts release S100A proteins. These molecules dimerize, forming homo- and heterodimers, which bind to various cell surface receptors. This binding activates several signalling pathways, altering the tumor microenvironment and promoting tumor progression and development. Individually, S100A13 is predominantly expressed in fibroblasts, osteoblasts, melanoma cells, and tumor astrocytes 5, 18, with localization in the plasma membrane, cytosol, and nucleoplasm 18. In the case of GBM, we observed relatively high expression of S100A13 in myeloid cells located at the tumor's leading edge. Furthermore, the expression of S100A13 does not seem to show a strong impact on the prognosis of IDH wt GBMs or IDH mut astrocytoma. A new view of the possible role of S100A13 in the glioma microenvironment of what had been described as an angiogenic factor 38. This protein can bind to the Cu^2+^ ion and RAGE receptors, thereby inducing cellular signalling 5. It also acts as a carrier molecule for fibroblast growth factor (FGF-1), promoting its release 38. It stands out for its possible role in tumor angiogenesis, although its function is still unclear 38.

Finally, S100A11 is expressed in cells like macrophages, oligodendrocytes, tumor cells, epithelial cells, and GSCs 5, 14. It is found in the cytosol, nucleoplasm, or extracellular space 5, 14. This protein has high affinity for binding to annexin A2 (ANXA2) 44 and RAGE receptors 5, 45, activating the Wnt/β-catenin signalling pathway, contributing to oncogenesis, metastasis, tumor progression and drug resistance 42, 46. Likewise, in pathological contexts, it participates in the chemotaxis and infiltration of TAM and regular T lymphocytes to the tumor area (**Supplementary Table 1**) 13, 46.

**Figure 9:**
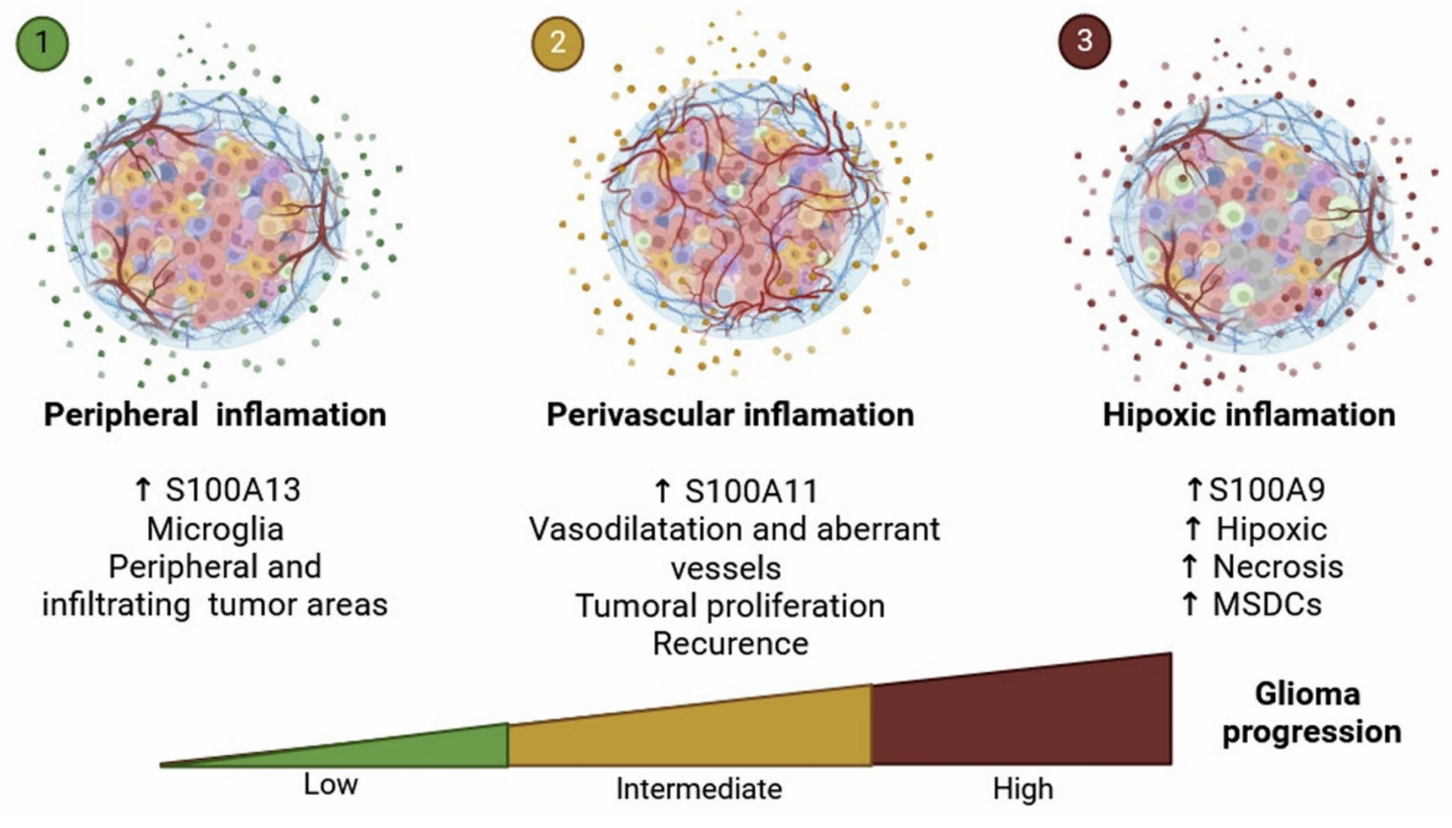
** Representative diagram of the different types of inflammation linked to glioma progression.** It shows the differential spatial distribution of S100A13, S100A11, and S100A9, allowing us to attribute specific functions to their locations within the architecture of glioblastoma.

We found a positive association between the expression of S100A9 and S100A11 and the enrichment of the various processes including angiogenesis, endothelial migration, cytotoxicity of T lymphocytes, activation of neutrophils, microglia and NK lymphocytes, cytokine production, chemotaxis of DC and cell-matrix adhesion (**Figure 9**). Conversely, S100A13 could be linked to the physiological function of microglia located in the periphery of gliomas and may play a more relevant role in the glioma invasion processes, irrespective of their aggressiveness (**Figure 9**). These findings indicate that these S100A proteins could be involved in the regulation and remodelling of the tumor microenvironment, contributing to the developed, progression and aggressiveness of glioma.

Because S100A9 and S100A11 showed a strong involvement in the aggressiveness of GBM and could have a direct effect on RAGE, the effect of Azeliragon on GBM tumor tissue was examined. It demonstrated a significant inhibitory effect on the perivascular inflammation signature in half of the GBM cases, while the hypoxic inflammation signature only had an effect in a quarter of GBMs. This could translate into a potentially important therapeutic effect for treating those GBMs that show inflammation-dependent growth. Thus, Azeliragon is currently being evaluated in two clinical trials, (1) in MGMT unmethylated Glioblastoma (NCT05986851), and (2) in combination with chemoradiotherapy in newly diagnosed Glioblastoma (NCT05635734).

In this study we have been able to delve deeper into the compression of S100A proteins in the MTE of glioblastoma. Our findings have managed to characterize the expression of the S100A9, S100A11 and S100A13 proteins in the GBM architecture, determining not only their cellular, histological and functional characteristics but also defining an inflammatory profile associated with them. Additionally, our research has managed to prove the therapeutic efficacy of a RAGE inhibitor, Azeliragon, through an innovative *ex vivo* assay of tumor fragments derived from the patient himself in parallel with its prospective phase I/II clinical trial (NCT05635734). In this sense, our results demonstrate that these proteins are suitable antitumor therapeutic targets, stratifying the various subtypes of gliomas based on their specific inflammatory profile.

## Materials and Methods

### Human samples

This study incorporated two retrospective cohorts of glioma patients (Table 1). The first cohort consists of 118 patients designated for the discovery analysis. The second cohort is a highly homogenized group of 13 patients who showed tumor recurrence. Every patient in this investigation was diagnosed at the “Hospital Universitario 12 de Octubre” in Madrid, Spain, between the years 2012 and 2023. All diagnoses were updated to align with to the current CNS WHO criteria of 2021. We collected both fresh-frozen and formalin-fixed paraffin-embedded (FFPE) tumor samples and we documented the clinic-pathological data, including age, gender, histological type of the tumor, treatment details, recurrence, and mortality status. Glioma tissue samples, either fresh-frozen or paraffin-embedded, were obtained were obtained after patient's written consent and with the approval of the Ethical Committees of “Hospital Universitario 12 de Octubre” (CEI 21/551; 24/084).

### Patient-derived tumor fragment (PDTFs)

For the preparation of PDTF cultures from GBM patients, we sectioned fresh tumor samples into approximately 5 mm slices. These slices were subsequently cultured in a collagen and matrigel blend as detailed in reference (32). After setting the initial culture, we treated/incubated them with Azeliragon (PF-04494700) at a concentration of 10 µg/ml for a 24-hour period. PDTFs will be grown in media complete media, DMEM (Thermo Fisher Scientific) supplemented with 10% FBS, penicillin-streptomycin at a 1:100 ratio, and growth factors, specifically EGF (40 ng/ml) and bFGF2 (20 ng/ml), procured from PeproTech. Following cultivation, the PDTFs were immediately frozen and stored at -80ºC, for subsequent transcriptional level analysis using qRT-PCR.

### qRT-PCR assay

RNA was extracted from the tissue employing RNA isolation Kit (Roche). The concentration and integrity of the harvested RNA were then evaluated spectrophotometrically at 260 nm. Total RNA (1µg) was reverse transcribed to cDNA with PrimeScript RT Reagent Kit (Takara). Quantitative real time PCR was performed using the Light Cycler 1.5 (Roche) with the SYBR Premix Ex Taq (Takara). The primers used for each reaction are indicated in Table 2 and Table 3. Gene expression was quantified by the delta-delta Ct method.

### Human

### Mouse

### Histological processing of tissue

Paraffin-embedded tumors were histologically sectioned into 5 µm slices using a microtome and collected on gelatinized slides for subsequent immunohistochemical or immunofluorescence staining.

### Immunofluorescent (IF) and immunohistochemical (IHC) staining

Histological sections were heated at 65ºC for 1 hour for deparaffinization and immersed in xylene, 100%, 96%, 70% ethanol and water sequentially for dehydration. Consecutively, the antigen was recovered in hot 10 mM sodium citrate pH6 followed by permeabilization of the membrane with 1x PBS and 1% Triton X-100 for 45 minutes. Nonspecific binding was then blocked by incubation with 1x PBS, 0.1% Triton X-100, and fetal bovine serum (FBS) at room temperature (RT) for 1 hour. Immediately after, incubation was carried out with the corresponding primary antibody (AC1º) (Table 4); in blocking solution at 4ºC overnight (o.n). On the subsequent day, sections were washed with PBS 1x and incubated with the appropriate secondary antibody (anti-mouse/rabbit-Dylight 488, anti-mouse/Rabbit-Cy3, anti-mouse/rat-Cy5, all from Jackson Immunosearch) (1:200 dilution) in RT and darkness for 2 hours. Finally, after washing, the slices stained with DAPI (2 µg/ml in 0.1 M PBS) and mounted using Fluoromount-G medium. The images obtained were made with the Leica SP-5 Thunder confocal microscope with the 20x and 40x objectives and were analysed with the ImageJ program. Otherwise, for immunohistochemistry, the slices were incubated with biotinylated secondary antibodies (1:200 dilution). Target proteins were visualized employing the ABC Kit combined with the DAB kit (Vector Laboratories). Once mounted, the sections were observed under a bright field microscope, with the obtained images being analyzed via ImageJ software, using different plugins like Diameter.

### *In silico* analysis

We accessed The Cancer Genome Atlas (TCGA) Firehouse Legacy dataset, including GBM, LGG, and GBM+LGG and Genotype-Tissue Expression (GTEx) dataset through cBioPortal (https://www.cbioportal.org/), and UCSC Xena-Browser (https://xenabrowser.net), to extract data on gene expression levels (RNAseq IlluminaHiSeq and UffyU133a), overall survival, and the distribution of the different genetic alterations, considering astrocytomas (A) IDH mut and glioblastomas (GBM) IDH wt. Using the expression values from each gene, we constructed Kaplan-Meier survival curves and stratified them into low and high expression groups. The significance of differences in survival between these groups was determined by employing the log-rank test (Mantel-Cox). For functionality studies, we utilized the "David Gene Ontology" analysis, consulting the PANTHER Classification System dataset. Initially, we identified a cluster of at least 500 genes co-expressed with the target gene (either S100A9, S100A11 or S100A13), based on the highest Spearman correlation values. The subsequent "David Gene Ontology" analysis linked the expression of these genes to associated biological processes. To assess gene signature enrichment across various tumor histological structures, we analysed the gene expression patterns (S100A9, S100A11 and S100A13) in different tumor areas (leading edge, infiltrating tumor cells, cellular tumor, necrotic zone and vascular zone), using IvyGAP dataset (Ivy Glioblastoma Atlas Project, http://glioblastoma.alleninstitute.org/). Based on the data obtained, we defined the signatures of perivascular and hypoxic inflammation. Furthermore, Receiver Operational Characteristics (ROC) analysis was performed to evaluate the diagnostic value of these signatures of genetic expression through calculating the sensitivity, specificity and are under the curve (AUC) in glioma.

Gene Set Enrichment Analysis (GSEA) pre-ranked was computed into the TCGA GBM cohort (RNAseq (IlluminaHiSeq)) using positive and negative co-expression of differentially expressed genes in IDH wt samples and GSEA software (GSEA). of genes, RRID:SCR_003199, v4.2.1) and gene collections were obtained from the cBioportal database (TCGA GBM cohort, Firehose Legacy) MSigDB (v7.5.1).

### Statistical analysis

For the statistical analysis of the samples, no specific statistical method was utilized to predetermine the sample size, but the sizes of our cohort are like or larger than those reported in previous publications 47-49. Due to the study's exploratory nature, neither randomization nor blinding was applied. The normality of the data distribution and the variance was formally tested and, therefore, non-parametric or parametric statistical analyses were used (two-tailed unpaired Student's t test, Wilcoxson-Mann Whitney or Welch´s correction). All statistical analyses were performed employing GraphPad Prism 8.0 software. Significance levels were denoted as *P < 0.05, **P < 0.01, ***P < 0.001, and ****P < 0.0001. SEM stands for Standard Error of the Mean. For Kaplan-Meier survival curves, the level of significance was determined by the two-tailed log-rank test. For correlation analysis between each gene, expression data were tested by Pearson's correlation coefficient. Precise experimental details are provided in the figure legends.

### Mouse glioma cells

GL261 murine glioma cells were maintained in DMEN plus 10% FBS supplemented with penicillin-streptomycin (1:100) (Lonza Group AG); 0.4% heparin (Sigma-Aldrich); and 40 ng/mL of EGF and 20 ng/mL of bFGF2 (Peprotech). Both cells were obtained from the NCI-Frederick Cancer Research Tumor Repository (Frederick, MD, USA).

### Intracranial tumor formation and in *in vivo* treatment

Animal experiments received approval from the Research Ethics and Animal Welfare Committee UAM and Comunidad de Madrid (PROEX 183.4/22) (Madrid, Spain), following both European Union and national guidelines. C57BL/6 adult mice were used for the experiment. Intracranial transplantation to establish orthotopic allografts was performed by injecting 7000 cells resuspended in 2 μL of stem cell culture medium using a Hamilton Syringe. Injections were precisely targeted to the striatum using a Stoelting Stereotaxic apparatus (coordinates: A-P, -0.5 mm; M-L, +2 mm; D-V, -3 mm relative to Bregma). Mice were sacrificed upon the appearance of symptoms. Treatment with Azeliragon included a daily intraperitoneal dose of this drug (0.2 mg/kg) for 25 days, starting from the third day of tumor implantation (day 0).

## Supplementary Material

Supplementary figures and table.

## Figures and Tables

**Table 1:** Summary of clinical features from our Glioma (n = 116) and Recurrence cohorts (n = 13).

Glioma Cohort	
Patient characteristics	n = 116
Median age (years)	54 years
Median KPS (range)	85 (70-100)
Gender	
Female	46 (39.66 %)
Male	70 (60.35 %)
Race	
Black or African American	0 (0 %)
White	116 (100 %)
Histological Type	
Astrocytoma	107 (92.4 %)
Oligodendroglioma	9 (7.76 %)
Grade	
Low grade glioma (II-III)	35 (30.17 %)
High grade glioma (IV)	81 (69.83 %)
Molecular status	
IDH mutated	35 (30.17 %)
MGMT methylated	40 (34.48 %)
Treatment	
Stupp protocol	89 (76.72 %)
**Glioma Recurrence Cohort**	
Patient characteristics	n = 13
Median age (years)	31 years
Median KPS (range)	80 (70-90)
Gender	
Female	4 (30.8 %)
Male	9 (69.23 %)
Race	
Black or African American	0 (0 %)
White	13 (100 %)
Histological type	
Astrocytoma	9 (69.23 %)
Oligodendroglioma	4 (30.8 %)
Grade	
Low grade glioma (II-III)	4 (30.8 %)
High grade glioma (IV)	9 (69.23 %)
Molecular status	
IDH mutated	13 (100%)
MGMT methylated	2 (15.38 %)
Treatment	
Stupp protocol	10 (79.62 %)

**Table 2:** Human qRT-PCR primers.

Gene	Forward sequence	Reverse sequence
GAPDH	5´-GTCTCCTCTGACTTCAACAGCG-3´	3´-ACCACCCTGTTGCTGTAGCCAA-5´
S100A9	5´-GCACCCAGACACCCTGAACCA-3´	3´-TGTGTCCAGGTCCTCCATGATG-5´
S100A11	5´-CCAGAAGTATGCTGGAAAGGATG-3´	3´-CATCATGCGGTCAAGGACACCA-5´
LGALS3	5´-CCATCTTCTGGACAGCCAAGTG-3´	3´-TATCAGCATGCGAGGCACCACT-5´
CAV1	5´-CCAAGGAGATCGACCTGGTCAA-3´	3´-GCCGTCAAAACTGTGTGTCCCT-5´
SERPINE1	5´-CTCATCAGCCACTGGAAAGGCA-3´	3´-GACTCGTGAAGTCAGCCTGAAAC-5´
CD163	5´-CCAGAAGGAACTTGTAGCCACAG-3´	3´-CAGGCACCAAGCGTTTTGAGCT-5´
ADAM8	5´-TGCTGGAGGTGGTGAATCACGT-3´	3´-TCAGGAGGTTCTCCAGTGTGAC-5´
CXCR4	5´-CTCCTCTTTGTCATCACGCTTCC-3´	3´-GGATGAGGACACTGCTGTAGAG-5´
CXCL8	5´-GAGAGTGATTGAGAGTGGACCAC-3´	3´-CACAACCCTCTGCACCCAGTTT-5´
SIGLEC7	5´-CTGGTCTTCCTCTCCTTCTGTG-3´	3´-GCATCCTTCATGCCTATGTCTCC-5´
SIGLEC9	5´-CCACGAACAAGACCGTCCATCT-3´	3´-TCTGGGAGTGACAGAGATGAGC-5´
ACSL1	5´-ATCAGGCTGCTCATGGATGACC-3´	3´-AGTCCAAGAGCCATCGCTTCAG-5´
CCL4	5´- GCTTCCTCGCAACTTTGTGGTAG-3´	3´-GGTCATACACGTACTCCTGGAC-5´
LILRB4A	5´-ACAAGGTCCGTCTCAACAGCTG-3´	3´-GAAGCAGGATGGAGACCACCAA-5´
NR4A3	5´-ACTGCCCAGTAGACAAGAGACG-3´	3´-GTTTGGAAGGCAGACGACCTCT-5´
WNT5A	5´-TACGAGAGTGCTCGCATCCTCA-3´	3´-TGTCTTCAGGCTACATGAGCCG-5´
TLR6	5´-ACTGACCTTCCTGGATGTGGCA-3´	3´-TGACCTCATCTTCTGGCAGCTC-5´
CX3CR1	5´-CACAAAGGAGCAGGCATGGAAG-3´	3´-CAGGTTCTCTGTAGACACAAGGC-5´
IL17RA	5´-CTGTATGACCTGGAGGCTTTCTG-3´	3´-CGAGTAGACGATCCAGACCTTC-5´

**Table 3:** Mouse qRT-PCR primers.

Gene	Forward sequence	Reverse sequence
Gapdh	5´-CATCACTGCCACCCAGAAGACTG-3´	3´-ATGCCAGTGAGCTTCCCGTTCAG-5´
S100A9	5´-TGGTGGAAGCACAGTTGGCAAC-3´	3´-CAGCATCATACACTCCTCAAAGC-5´
S100A11	5´-GAAGGATGGAAACAACACTCAACT-3´	3´-CGTCACAGTTGAGGTCCAGCTT-5´
Lgals3	5´-AACACGAAGCAGGACAATAACTGG-3´	3´-GCAGTAGGTGAGCATCGTTGAC-5´
Cav1	5´-CACACCAAGGAGATTGACCTGG-3´	3´-CCTTCCAGATGCCGTCGAAACT-5´
Serpine1	5´-CCTCTTCCACAAGTCTGATGGC-3´	3´-GCAGTTCCACAACGTCATACTCG-5´
Cd163	5´-TCACTCCTGGGCTGCACGTAAAC-3´	3´-GATGTTATTTGCCATACAGGAGAATT-5´
Adam8	5´-TGCCAACGTGACACTGGAGAAC-3´	3´-GCAGACACCTTAGCCAGTCCAA-5´
Cxcr4	5´-GACTGGCATAGTCGGCAATGGA-3´	3´-CAAAGAGGAGGTCAGCCACTGA-5´
Cxcl15	5´-GGTGATATTCGAGACCATTTACTG-3´	3´-GCCAACAGTAGCCTTCACCCAT-5´
Siglec-e	5´-GTGTCCACAAGAATGACCATCCG-3´	3´-TGAGCCATTCTTCAGGATTGTGG-5´
Siglec-3	5´-GCATCTGATGCTGTGACTCCAG-3´	3´-AGTGTGGACACTGCTCTGTTCC-5´
Ascl1	5´-CGGAACTGATGCGCTGCAAACG-3´	3´-GGCAAAACCCAGGTTGACCAAC-5´
Ccl4	5´- ACCCTCCCACTTCCTGCTGTTT-3´	3´-CTGTCTGCCTCTTTTGGTCAGG-5´
Lilrb4a	5´-CTGGATGCTGTTACTCCCAACC-3´	3´-TGGGTGTAGAGGACTGGTCCTT-5´
Nr4a3	5´-ACGCCGAAACCGATGTCAGTAC-3´	3´-CTCCTGTTGTAGTGGGCTCTTTG-5´
Wnt5a	5´-GGAACGAATCCACGCTAAGGGT-3´	3´-AGCACGTCTTGAGGCTACAGGA-5´
Tlr6	5´-GTGAGGATGCTGTGTCAGTGGA-3´	3´-CCAGGCAGAATCATGCTCACTG-5´
Cx3cr1	5´-GAGCATCACTGACATCTACCTCC-3´	3´-AGAAGGCAGTCGTGAGCTTGCA-5´
Il17ra	5´-AGTGTTTCCTCTACCCAGCAC-3´	3´-GAAAACCGCCACCGCTTAC-5´

**Table 4:** Primary antibody.

Technique	Primary antibody	Antibody	Source	dilution
IF	S100A9	Rabbit	CellSignaling (#34425)	1:200
	CD68	Mouse	DAKO (M0876)	1:100
	S100A11	Rabbit	Abcam (ab180593)	1:400
	α SMA	Rabbit	Santa Cruz (sc-32251)	1:200
	S100A13	Rabbit	Atlas Antibodies (HPA019592)	1:500
	P2RY12	Rabbit	Atlas Antibodies (HPA014518)	1:500
IHC	S100A9	Rabbit	CellSignaling (#34425)	1:800
	S100A11	Rabbit	Abcam (ab180593)	1:400
	S100A13	Rabbit	Atlas Antibodies (HPA019592)	1:500
	GLUT1	Mouse	Santa Cruz (sc-377228)	1:200
	CD34	Mouse	Leica (NCL-L-END)	1:100
	MIB-1/KI67	Mouse	Santa Cruz (M7248)	1:500
	P2RY12	Rabbit	Atlas Antibodies (HPA014518)	1: 1000
