# Supplementary material for: S100A proteins show a spatial distribution of inflammation associated with the glioblastoma microenvironment architecture
Source: Theranostics. 2025 Jan 1;15(2):726–44. doi: 10.7150/thno.100638 (PMC11671380; doi:10.7150/thno.100638)
Supplement: Supplementary file 1 — Supplementary figures and table. [file thnov15p0726s1.pdf]

SUPPLEMENTAL MATERIAL

Characteristics	S100A9	S100A11	S100A13
Phenotype	Immunosuppressive	Tumor	Angiogenic
Receptor	RAGE, TLR4, GPCR, CD36, heparan sulfate, N-glycans, CD147 or EMMPRIM, SR	RAGE, ANXA2	RAGE
Subcellular location	Plasma membrane, cytosol and intermediate filaments	Cytosol, nucleoplasm	Plasma membrane, cytosol, nucleoplasm
Cellular expression	Monocytes, neutrophils, DCs, macrophages, fibroblasts, tumor cells, endothelial cells and keratinocytes	Oligodendrocytes, macrophages, tumor cells, epithelial cells	Fibroblasts, osteoblasts and melanoma cells
Expression in CSCs	Yes	Yes	
Tumor location			Perivascular regions.
Immune cell associated	Neutrophils<br>Monocytes<br>PBMC<br>DC	Neutrophils<br>Basophils<br>Eosinophils<br>PBMC<br>Monocytes<br>T, B, NK lymphocytes	NK cells<br>DC<br>B and T lymphocytes, monocytes
Chemotaxis	From monocytes, TAM and TAN	From TAM and Treg lymphocytes	
Hypothetical functions	Proliferation and cell cycle, differentiation, motility, invasion and migration, apoptosis	Proliferation and cell cycle, differentiation, apoptosis	Mobility, invasion and migration
Pathways	It activates NF-KB and MAPK, STAT, PI3K/AKT/mTOR pathways, promoting carcinogenesis, tumor progression, angiogenesis, and drug resistance.	It activates the Wnt/β-catenin pathway, promoting metastasis and drug resistance.	
Others	It is secreted by necrotic or immune cells activated in pathological conditions.	It is hypomethylated gene in GBM	It is a cargo FGF-1 molecule<br>It can bind to Cu^{2+}

Supplementary Table 1. Summary of features of S100A9, S100A11, S100A13

SUPPLEMENTARY FIGURES

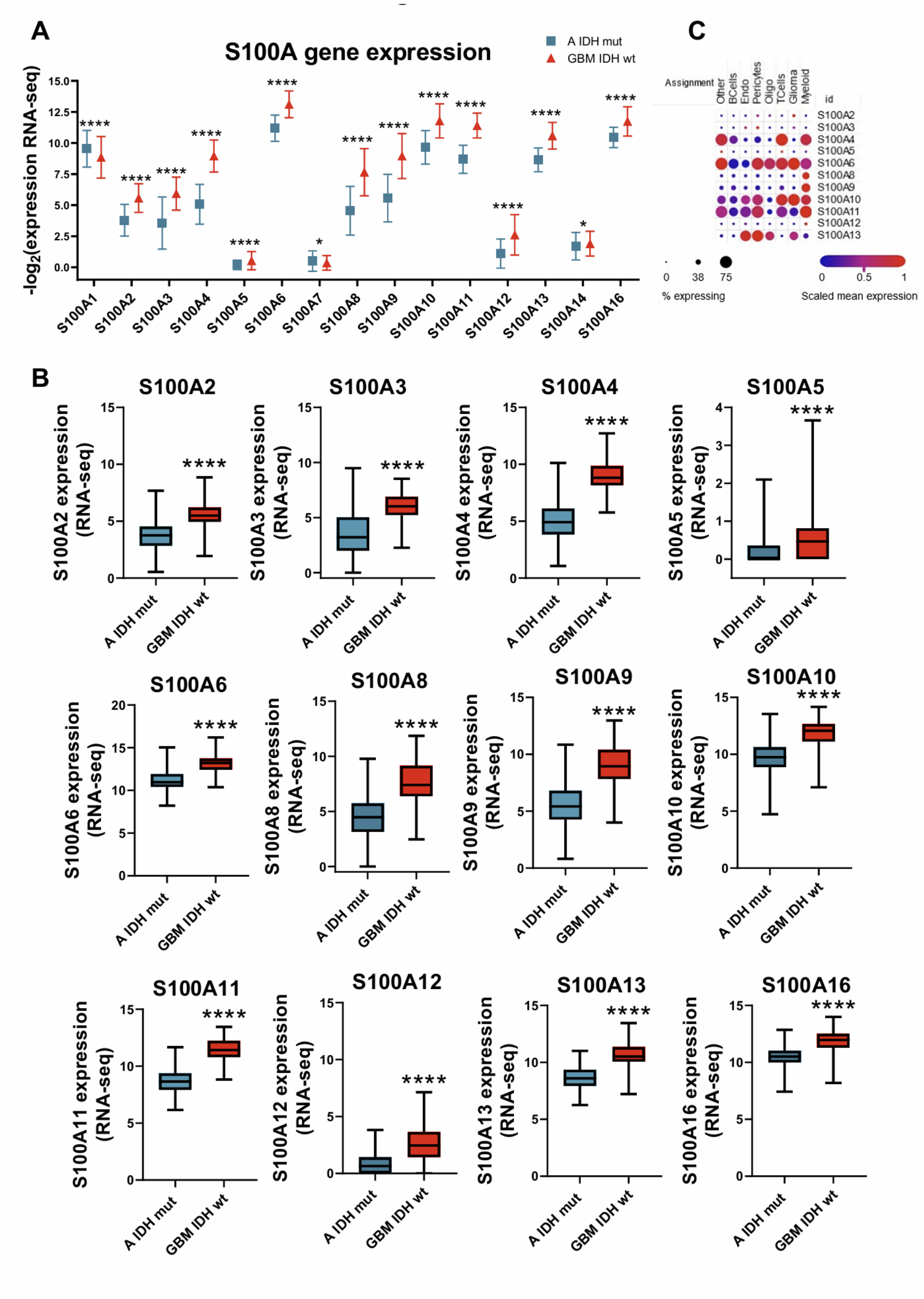

Supplementary Figure 1. Association between the overexpression of some S100A genes and cellular heterogeneity in patients with glioma. (**A-B**) Boxplot of S100A expression comparing IDH mut astrocytoma brain samples (n = 404) versus IDH wt GBM samples (n = 143) (TCGA-Merge-LGG+GBM cohort). (**C**) scRNA-seq analysis of the expression levels of of S100A genes in glioma cells. Data represent mean ± SD. **** P ≤ 0.001 * P ≤ 0.05; n.s., not significant. Statistical significance was determined by Mann Whitney test.

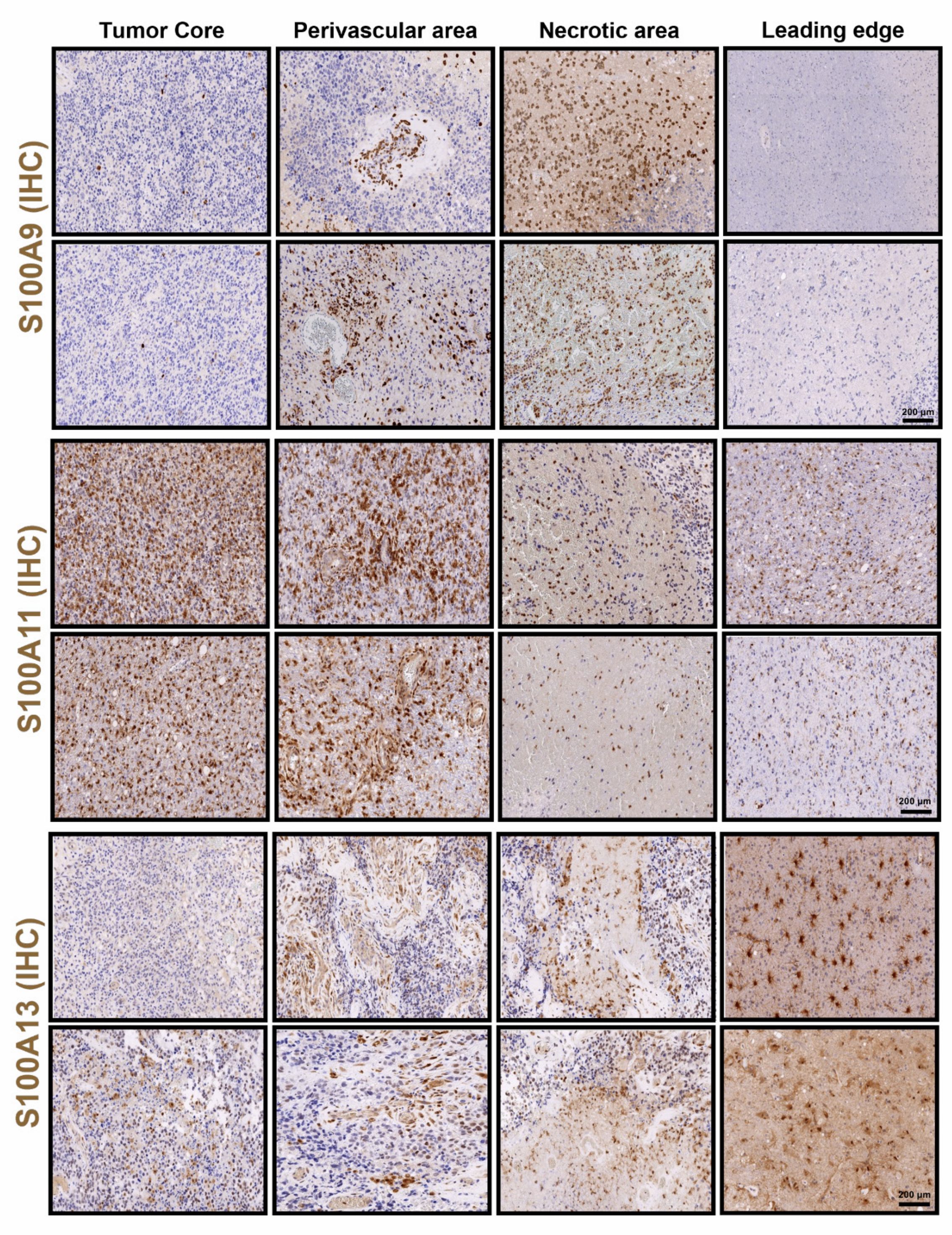

Supplementary Figure 2. Histological distribution of S100A9, S100A11 and S100A13 proteins in GBM. (**A-C**) Representative IHC images of S100A9 (A), S100A11 (B), S100A13 (C) expression in different regions of GBM samples, including the tumor core, vascular and necrotic areas, and the leading edge. Scale bar 200 µm.

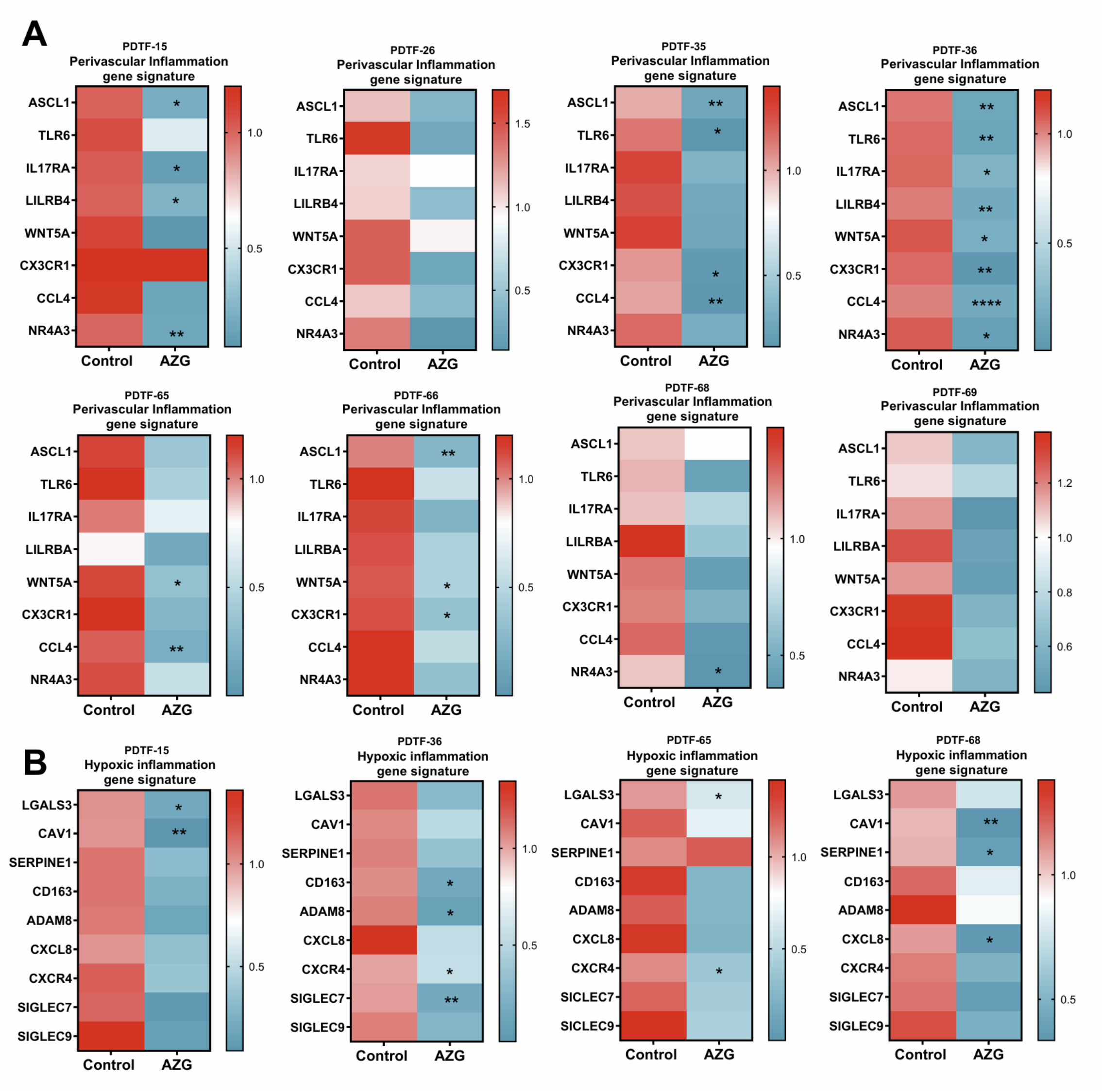

Supplementary Figure 3. Treatment with Azeliragon in PDTFs led to a response linked to perivascular and hypoxic inflammation. (**A-B**) Comparison of the quantification of gene expression of the signature of perivascular inflammation after treatment with AZG (10 µg/ml for 24 h) in all PDTFs analysed. Data represent mean ± SD. *** P ≤ 0.001; ** P ≤ 0.01; * P ≤ 0.05; n.s., not significant. Statistical significance was determined by Student’s t-test. PDTF; Patients Derived Tumor Fragment; AZG; Azeliragon.

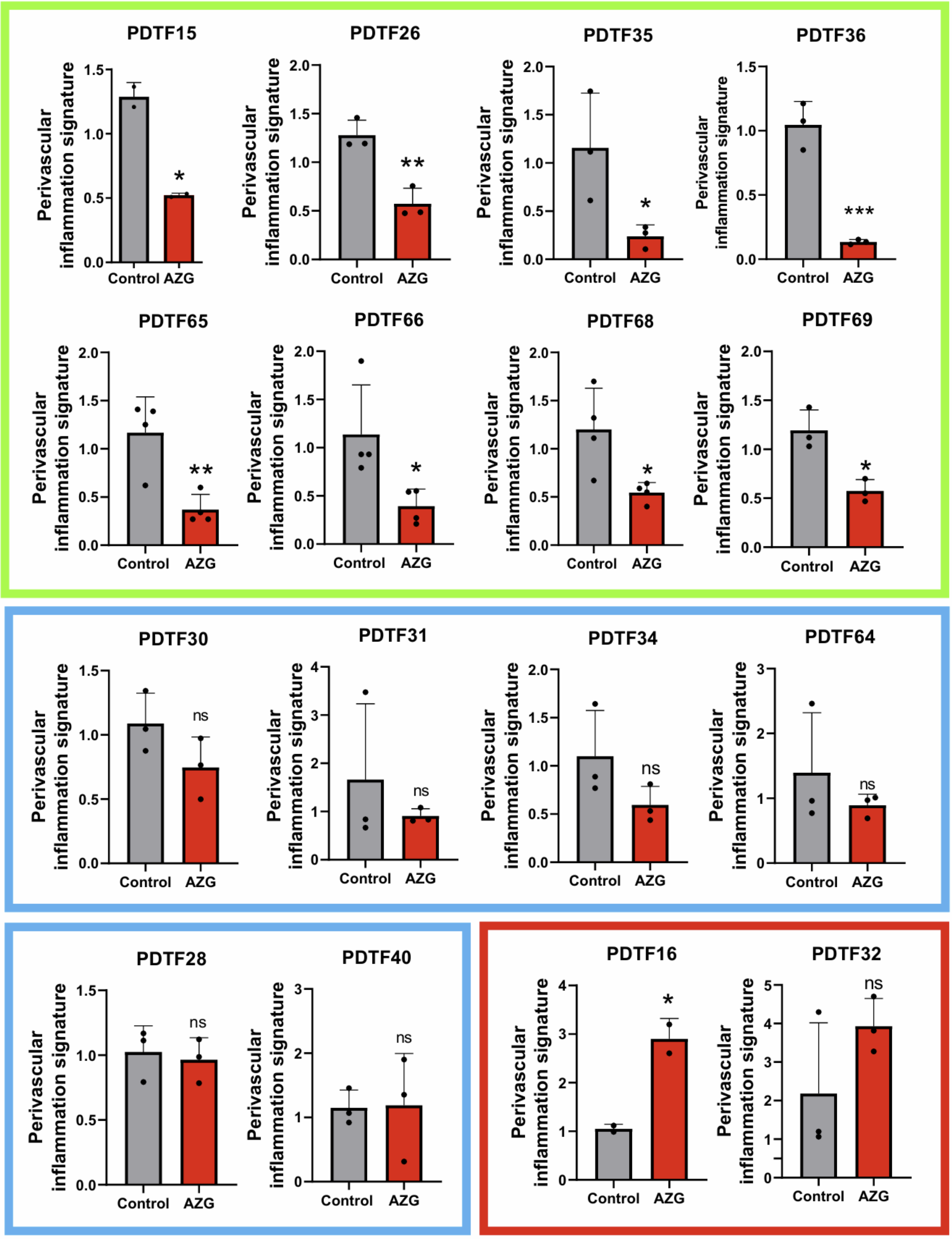

Supplementary Figure 4. Treatment with Azeliragon in PDTFs led to a response linked to perivascular and hypoxic inflammation. (**A-B**) Comparison of the quantification of gene expression of the signature of hypoxic inflammation after treatment with AZG (10 µg/ml for 24 h) in all PDTFs analysed. Data represent mean ± SD. *** P ≤ 0.001; ** P ≤ 0.01; * P ≤ 0.05; n.s., not significant. Statistical significance was determined by Student's t-test. PDTF; Patients Derived Tumor Fragment; AZG; Azeliragon.

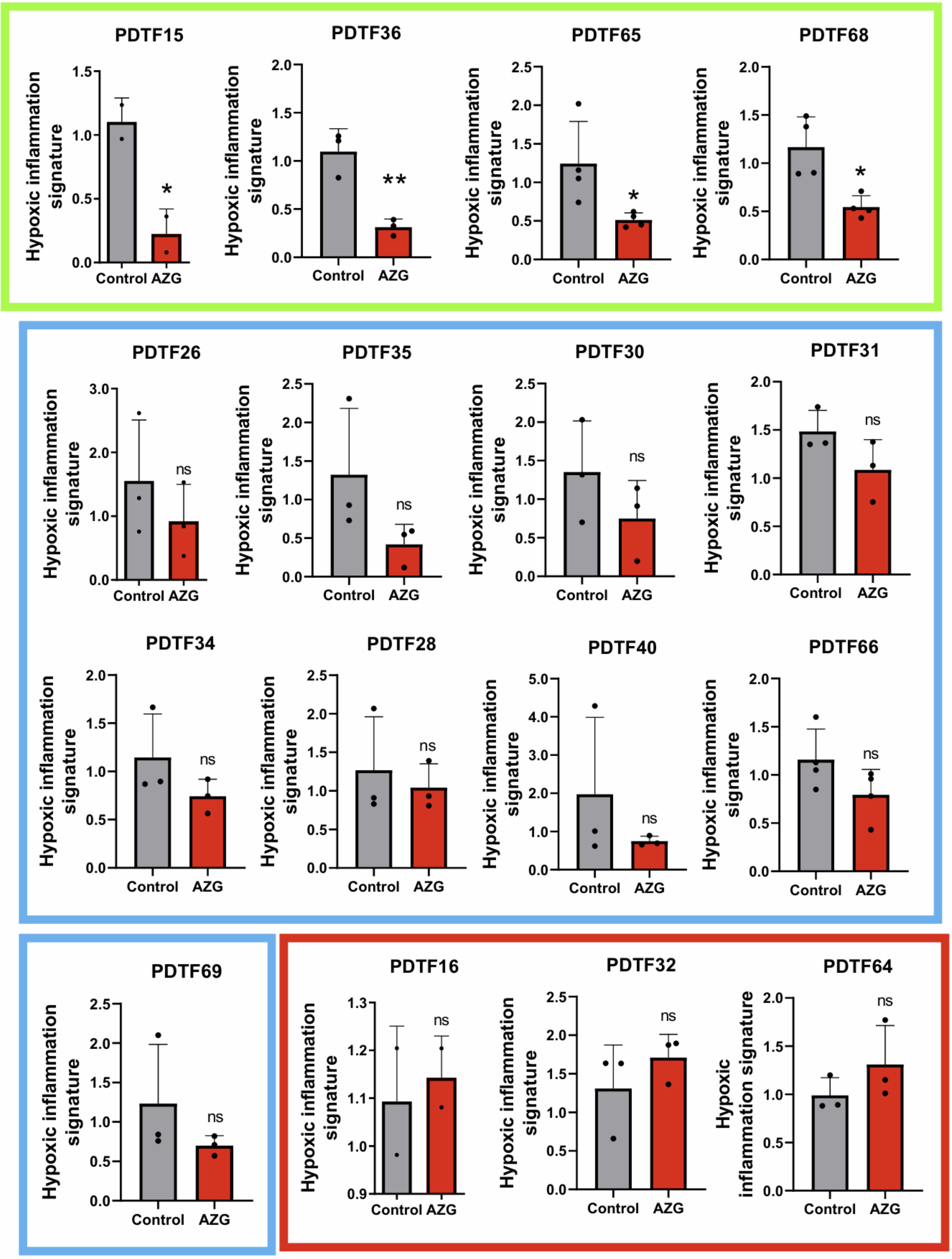

Supplementary Figure 5. AZG generated a significantly greater decrease in the signature of perivascular inflammation compared to that of hypoxic inflammation. (A-B) Heat maps of the expression of the genetic signature of perivascular (A) and hypoxic (B) inflammation comparing AZG treatment and the control condition (vehicle). Data represent mean ± SD. **** P ≤ 0.0001; *** P ≤ 0.001; ** P ≤ 0.01; * P ≤ 0.05; n.s., not significant. Statistical significance was determined by Student's t-test. PDTF; Patients Derived Tumor Fragment; AZG; Azeliragon.
